# Theoretical study of metal-free catalytic for catalyzing CO-oxidation with a synergistic effect on P and N co-doped graphene

**DOI:** 10.1038/s41598-022-14286-8

**Published:** 2022-06-21

**Authors:** Sarinya Hadsadee, Siriporn Jungsuttiwong, Rui-Qin Zhang, Thanyada Rungrotmongkol

**Affiliations:** 1 Center of Excellence in Biocatalyst and Sustainable Biotechnology, Department of Biochemistry, Faculty of Science, Chulalongkorn University, Bangkok, 10330 Thailand; 2 Department of Chemistry and Center of Excellence for Innovation in Chemistry, Ubon Ratchathani University, Ubon Ratchathani, 34190 Thailand; 3 Department of Physics and Materials Science and Centre for Functional Photonics (CFP), City University of Hong Kong, Hong Kong, China; 4 Program in Bioinformatics and Computational Biology, Graduate School, Chulalongkorn University, Bangkok, 10330 Thailand

**Keywords:** Chemistry, Materials science

## Abstract

P and N co-doped graphene (PN_x_C_y_-G with x = 1, 2, 3 and y = 0, 1, 2) is designed to enhance graphene reactivity with a synergistic effect of the P and N atoms for the CO oxidation reaction, focusing on the influence of the N dopant concentration on graphene. The calculated results indicate that increasing two or three coordinated N to P can facilitate charge transfer from the surface onto O_2_ molecules. However, the adsorbed O_2_ molecule breaks apart on **PN**_**3**_**-G** surface, affecting CO oxidation performance. Furthermore, **PN**_**2**_**C**_**1**_**-G** exhibits excellent catalytic activity towards the oxidation of CO via the ER mechanism, which catalyzes CO oxidation with the rate-determining step of only 0.26 eV for the first and 0.25 eV for the second oxidation at 0 K. Additionally, the catalytic oxidation of **PN**_**2**_**C**_**1**_**-G** via Eley–Rideal mechanism prefers to occur at room temperature (298.15 K), with a rate-determining step of 0.77 eV. The reaction rates at 298.15 K is calculated to be 5.36 × 10^16^ mol s^–1^. The rate constants are obtained according to harmonic transition state theory, which could be supportive for catalytic oxidation of CO on the experiment.

## Introduction

Carbon monoxide (CO) is a well-known air pollutant^1^. Generally, CO gass occurs from the combustion processes of industry, factories, and incomplete combustion by gasoline and diesel-fueled engines. Importantly, it causes dangerous effects when we breathe CO which is harmful to the heart and brain. Therefore, removing this toxic gas are essential for environmental safety. The conversion of CO to carbon dioxide (CO_2_) is a desirable method in heterogeneous catalysis. Although CO_2_ is a greenhouse gas responsible for global warming, it is not hazardous to human health.

The catalytic oxidation of CO has been studied to find an efficient catalyst to control the pollutant^2,3^. The CO oxidation reaction route involves the direct oxidation of CO to CO_2_ by oxygen (O_2_) adsorbed on the surface of a catalyst^4^. Previously, various noble metals, such as Pt, Pd, Cu, Fe, Rh, and Au were investiged for the development of a catalyst for CO oxidation^5–7^. Such catalysts are highly active toward the oxidation of CO; however, noble metals are rare and expensive. Moreover, these metal catalysts usually operate at a high reaction temperature. Thus, it is of great interest to develop efficient and low-cost catalysts for the low-temperature operation of the CO oxidation reaction. Metal-free catalysts have attracted attention due to their high activities in the catalytic oxidation reaction.

Various kinds of carbon-based materials, such as carbon nanotubes and graphene, have been studied to search for a metal-free catalyst for the oxidation of CO. Graphene is interesting material because of its unique properties deriving from a two-dimensional layered structure of sp^2^-hybridized carbon. Using sp^2^-hybridized carbon atoms to form hexagons is the focus of intensive investigation due to their significant physical and chemical properties. In particular, the high surface area, high chemical stability, and outstanding conductivity of graphene make it an ideal support for metal atoms and clusters in making novel carbon–metal nanocomposite catalysts^8–12^. Besides, vacancy defects on graphene could enhance the binding and dispersion of both metals and metal-free cataysts. Recent studies have shown that the doping with heteroatoms on defective graphene effectively modifies its characteristics and improves stability in catalytic applications. As a result of comparing supported metal and non-metal catalysts, the supporting metals indicate their practical and high activity attributes due to the strong interaction between metals. The supporting substrate modify the charge redistribution and affects the reactive performance of the catalyst^13^. However, the high surface free-energy of metals promotes the formation of the metals into large clusters, and these aggregations affect the catalytic efficiency of a catalyst^14,15^. Therefore, the substitutional doping of metal-free atoms in the graphene surface is important to adapt the electronic distribution of the graphene system and promote catalyst performance. Additionally, chemically modified graphenes featuring metal-free substituents such as B, N, S and P^16–18^ were reported. The incorporation of a non-metal heteroatom into the graphene lattice is especially a promising approach to improve catalytic activity further. Particularly, N-doped graphene has been attracting considerable attention in theoretical and experimental studies. N-doped graphene is a non-precious metal catalyst for oxygen reduction reactions (ORRs). Additional electrons are introduced into graphene, conferring novel electronic properties by N-doping. Previously, Chang et al.^19^ demonstrated that B-N and P-N co-doped graphenes exhibited greater catalytic activity to reduce O_2_ than singly doped N-graphene. In addition, doping by B and P in graphene considerably modifies the electrophysical character of graphene due to the significant electronegativity difference between the B and N atom or P and N atoms, and this difference induces heterogeneity in the graphene surface. Liang et al.^20^ has also reported that P and N co-doped graphene improves the catalytic ability to reduce O_2_ due to a synergistic effect, compared to single doping. To the best of our knowledge, no experimental or theoretical study reports are published on the catalytic CO oxidation reaction over P and N co-doped on single vacancy P-embedded graphene. However, P and N doped graphene was synthesized and applied to ORRs. This co-doping strategy will allow the graphene based metal-free catalysis being effective in the CO oxidation reaction. The focus of this study is to examine the synergic effect of doped P and N atoms for oxidation of CO by O_2_ and to reveal how incorporating an N atom around P can improve the catalytic activity of the surface, the adsorption configuration, and the electronic structure over P and N co-doped graphene. The effect of N dopant concentration on single vacancy P-embedded graphene for the reaction of CO oxidation. Furthermore, all possible reaction pathways for CO oxidation reaction are investigated via density functional theory (DFT).

### Computational details

Calculations were performed using the DMol^3^ software package in Materials Studio 7.0^21^ with the Perdew–Burke–Ernzerhof (PBE)^22^ functional in the generalized gradient approximation (GGA). The wave function for all atoms was described in terms of a double numerical basis set with polarization (DNP)^23^. We used the DFT + D method within the Grimmeʼs scheme^24,25^ to consider the Van der Waals effects. No symmetry constraint was employed during the geometry optimizations. All geometry optimizations were performed using a convergence tolerance of 1.0 × 10^−5^ Ha, a maximum force of 0.001 Ha/Å, and a maximum displacement of 0.005 Å. To achieve accurate electronic convergence, a smearing of 0.005 Ha and a basis-set cut-off of 4.2 Å were employed.

A 5 × 5 supercell of graphene (containing 50 C atoms) was built as a base material. The vacuum space of 20 Å was set in the z-direction to avoid interactions between periodic images. The Brillouin zone (BZ) integration was sampled using a 5 × 5 × 1 k point. The transition state (TS) of CO oxidation reactions was serached using a linear synchronous transit (LST)/quadratic synchronous transit (QST) method to find the minimum-energy pathway (MEP) for each reaction step. TS optimization computations confirmed the connections of reacant and product to the TS. In addition, the TS was verified by vibrational frequencies, which guaranteed only one imaginary frequency throughout the potential energy surface. The density of states (DOS) was carried out with k-point grid of 15 × 15 × 1. To further ensure the stability of the catalyst, the molecular dynamics (MD) simulation of **PN**_**x**_**C**_**y**_**-G** was carried out for 2.0 ps in the NVT ensemble, with a 2.0 fs time step at 300 K. The Nosé-Hoover chain method^26^ was used to calculate the thermodynamic stability. The following formula was used to obtain the formation energy (E_f_) of **PN**_**x**_**C**_**y**_**-G**:

1 $$E_{f} = \left( {E_{PNxCy - G} - \mu_{P} - n\mu_{N} + m\mu_{C} } \right) - E_{G}$$

where E_PNxCy-G_ is the total energy of the graphitic **PN**_**x**_**C**_**y**_**-G** sheet, and E_G_ is the total energy of the pristine graphene. The µ_P_, µ_N_, and µ_C_ are the chemical potentials of P, N, and C atoms, respectively. The x and y parameters are the number of N and C atoms on the graphene sheet. The interaction between gas molecule and surface was studied by calculating the adsorption energy (E_ads_), which is defined by Eq. (2):

2 $$E_{ads} = E_{adsorbate/catalyst} - E_{catalyst} - E_{adsorbate}$$

where E_adsorbate/catalyst_ is the total energy of the adsorbate–catalyst system, E_catalyst_ the energy of the optimized **PN**_**x**_**C**_**y**_**-G** surface, and E_adsorbate_ the energy of a free atom. By this definition, a negative E_ads_ value represents an exothermic adsorption.

Next, the reaction rate constants of reaction step were carried out. The microkinetic simulation of the best CO oxidation reaction pathways were performed by using the MKMCXX software package^27^. The rate constants for the forward and backward elementary reaction were calculated by the Eyring equation:

3 $$k=Aexp\left(\frac{-Ea}{{k}_{b}T}\right)$$

where E_a_, *k*, *k*_b_ and T are activation barrier, the reaction rate constant, Boltzman constant and temperature in Kelvin, respectively. Here, the pre-factor A of the Eyring equation can be determined by

4 $$A=\frac{{k}_{bT}}{h}\frac{{Q}^{TS}}{Q}$$

where *h,* Q^TS^ and Q are the Planck constant, the transition state partition fiction and the initial state partition fiction, respectively. The partition fiction concluded all possible states, inclusive translation, rotation and vibration modes. In this study, the pre-factor A was set to be 10^13^ s^-1^ for the elementary surface reactions due to the negligible changes in entropy.

The non-activated molecular adsorption, gas-phase pressure was set to be 1 atm and the gas includes O_2_, CO and CO_2_. The gas surface and desorption rates were depicted by the Hertz–Kunden equation^28^.

5 $$F=S\frac{P}{\surd 2\pi m{k}_{b}T}$$

Thus, the gas adsorption rates constant (*K*_*ads*_) and desorption rates constant (*K*_*des*_) were provided by following,

6 $${K}_{ads}=S\frac{PA}{\surd 2\pi m{k}_{b}T}$$

and

7 $${K}_{des}=A\frac{{k}_{b}{T}^{3}}{{h}^{3}}\frac{2\pi {k}_{b}}{\sigma {\theta }_{rot}}\mathrm{exp}(\frac{-{E}_{des}}{RT})$$

where *A*, *m*, σ, *ϴ*_rot_, *S* and *P* indicate the area of surface site of adsorption, the mass of molecule, the symmetry number, the characteristic temperature for the rotation, the sticking coefficient (assumed as 1), Three rotational degrees of freedom and two translational degrees of freedom of transition state were assumed for desorption.

## Results and discussion

### Properties and stability of PN_x_C_y_-G graphene sheets

**Figure 1:**
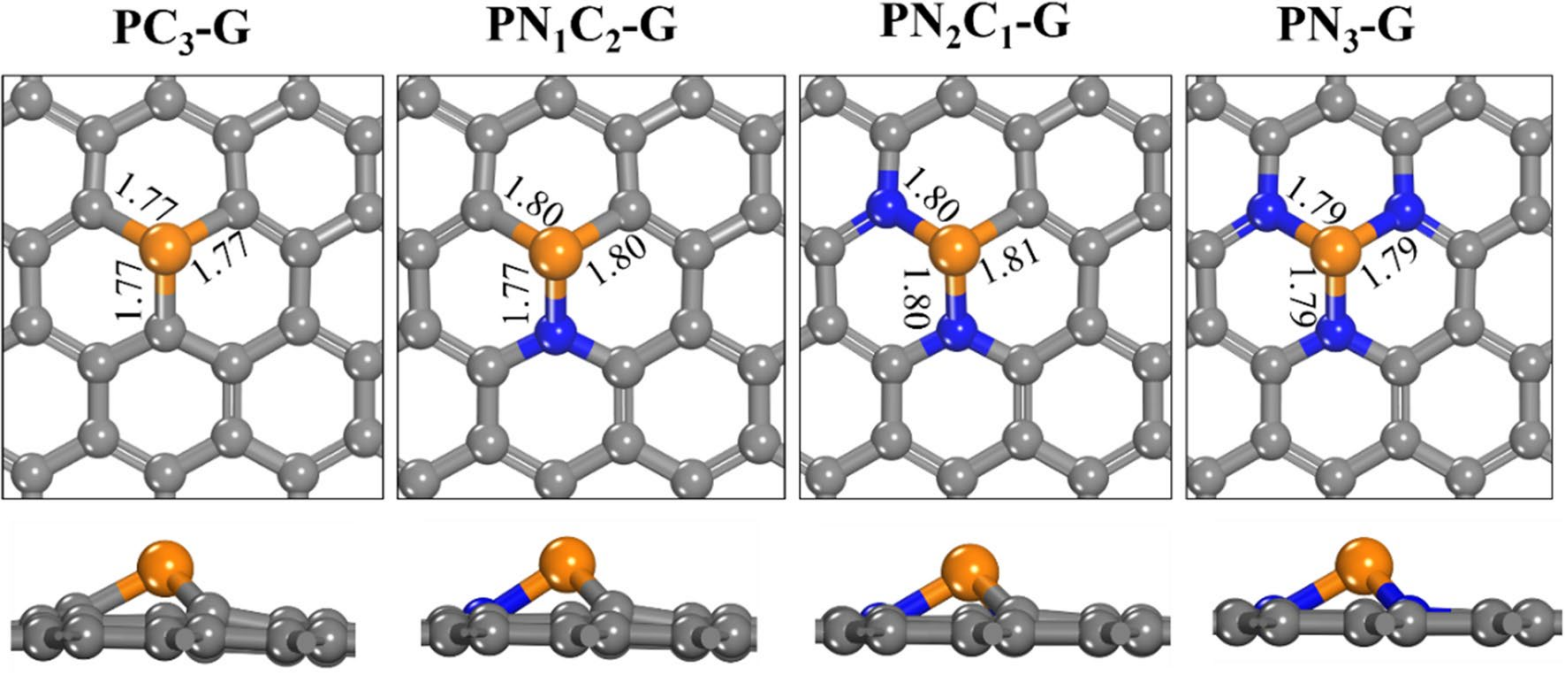
Optimized structures **PC**_**3**_**-G**, **PN**_**1**_**C**_**2**_**-G**, **PN**_**2**_**C**_**1**_**-G**, and **PN**_**3**_**-G**. All bond distances are in Å. Color scheme: C, gray; N, blue; P, orange.

We first optimized the structure of N doped on single vacancy P-embedded graphene (**PN**_**x**_**C**_**y**_**-G**). Figure 1 shows the optimized structure of **PN**_**x**_**C**_**y**_**-G** sheets where x and y = 0, 1, 2, and 3. The calculated atomic charge on the P atom and the formation energy of each graphitic sheet are summarized in supplementary Table 1. The calculated results show that after optimization, the structure of N and P co-doped in **PN**_**x**_**C**_**y**_**-G** is displaced outward from the graphene surface because of the larger atomic radius of P compared to C and N atoms. The bond distances between P and N in **PN**_**1**_**C**_**2**_**-G, PN**_**2**_**C**_**1**_**-G,** and **PN**_**3**_**-G** sheets are about 1.77, 1.79, and 1.79 Å, respectively, which are slightly longer than the P–C bond in the P–G graphene sheet (1.77 Å). The formation energies for **PN**_**1**_**C**_**2**_**-G, PN**_**2**_**C**_**1**_**-G,** and **PN**_**3**_**-G** are calculated as −1.90, −1.06, and −0.74, respectively. The result shows that the formation energy of **PN**_**1**_**C**_**2**_**-G** is the most negative. Therefore, co-doping of P and one N atom is more energetically favorable for P and N doping on a graphene surface.

**Figure 2:**
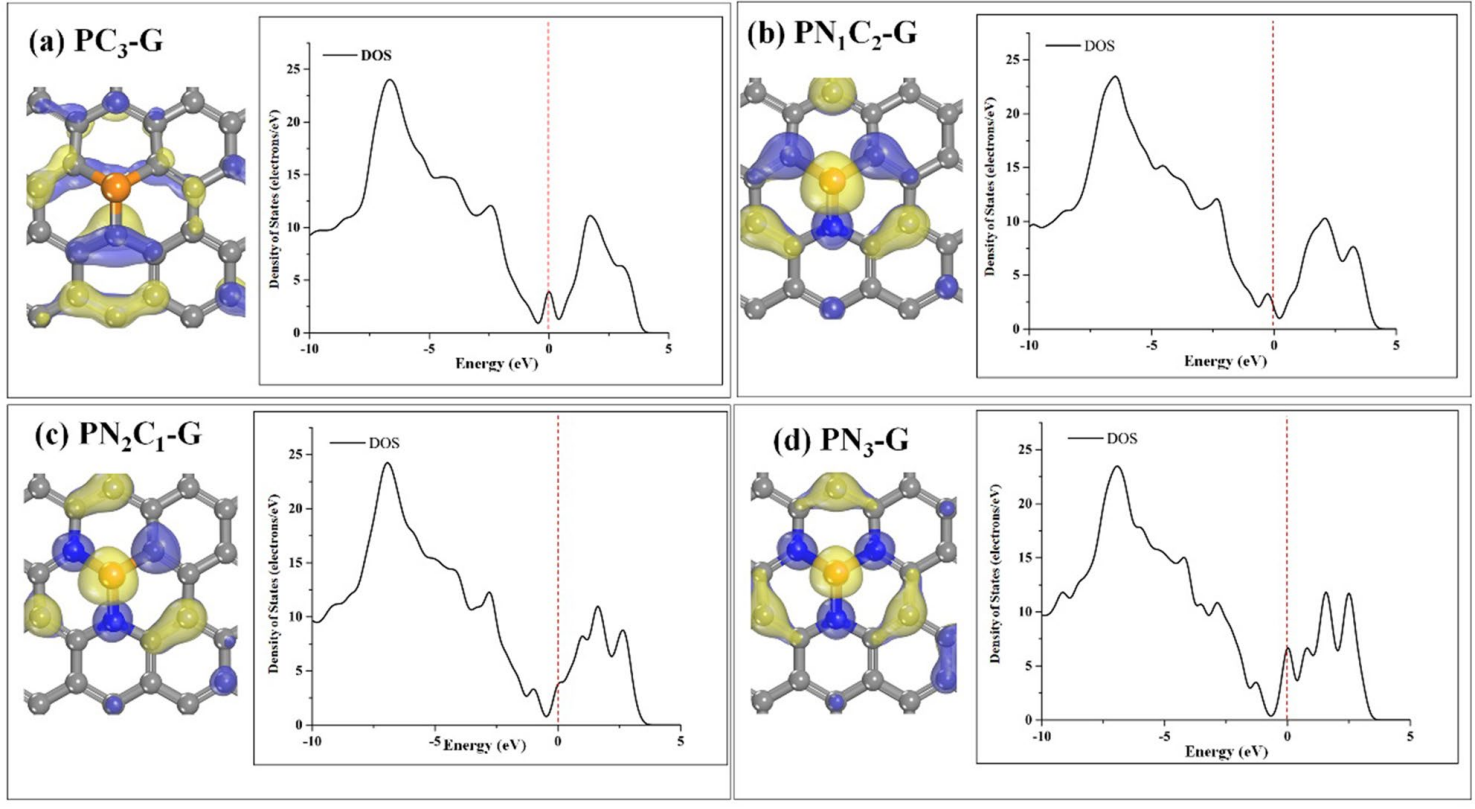
The highest occupied molecular orbital (HOMO), (the isovalue: ± 0.03) and the corresponding DOS plots of (**a**) **PC**_**3**_**-G**, (**b**) **PN**_**1**_**C**_**2**_**-G**, (**c**) **PN**_**2**_**C**_**1**_**-G**, and (**d**) **PN**_**3**_**-G** monolayers. The dashed line in the DOS plots indicates Fermi level (E_F_) which is set to zero energy.

Moreover, we also considered the stability of **PN**_**x**_**C**_**y**_**-G**, which is associated with its electron distribution. As shown in Fig. 2, the highest occupied molecular orbital (HOMO) displays differences in electronegativity between P, N, and C atoms, resulting in a local redistribution of charge density and electron aggregation on the highly electronegative N atoms. The blue and yellow in the deformed electron density map illustrate the capture and release of electrons, respectively. It is clearly seen that HOMOs are mainly distributed on P, N and C, implying that the surfaces are promoted by N atom. Due to the charges of the P and C atoms with weaker electronegativities are also affected by the size of the vacancy. In contrast, the electron densities of HOMO are redistributed for **PC**_**3**_**-G**, in which a single vacancy P is embedded graphene (Fig. 2a, left). According to supplementary Table 1, a P atom exhibits positive charges in **PC**_**3**_**-G** (0.638 |e|), **PN**_**1**_**C**_**2**_**-G** (0.692 |e|), **PN**_**2**_**C**_**1**_**-G** (0.810 |e|), and **PN**_**3**_**-G** (0.903 |e|) due to the transferring electrons that move from the P atom to neighboring atoms. Therefore, this charge causes P atom to form covalent bonds with coordinated C and N atoms. Additionally, partial density of states (PDOS) also supports strong interaction of C, N and P atoms on graphene surface (Supplementary Fig. 1). The results indicate that P atom and neighborhood atoms show the strong hybridization between P-3*p* and neighborhood atoms 2*p*-orbitals.

To gain deeper insights into the electronic structure of surfaces, we further calculated their density of states (DOS) and band structures. The results of the DOS plots reveal that the addition of a N atom to **PC**_**3**_**-G** leads to DOS near the Fermi level for **PN**_**1**_**C**_**2**_**-G** (Fig. 2b, right) whose valence band is close to Fermi level, indicating semi-metallic behavior. As showing in Fig. S2b, the valence band of **PN**_**1**_**C**_**2**_**-G** slightly shifts down from Fermi level. For **PC**_**3**_**-G** with 2 and 3 N-coordinations (Fig. 2c, d, right), we observe the the conduction band is shifted near Fermi level position of **PN**_**2**_**C**_**1**_**-G** and **PN**_**3**_**-G,** respectively. As presented in supplementary Fig. 2c, d for the band structures, the conduction band clearly shifts down to Fermi level. Thus, amount of coordinated N atom on graphene surface affect to electronic properties of surface. The **PN**_**1**_**C**_**2**_**-G** surface, doping N atoms impact holes (acceptor states), whereas for the **PN**_**2**_**C**_**1**_**-G** and **PN**_**3**_**-G** surfaces, doping N atoms affect electrons (donor states).

Therefore, **PN**_**2**_**C**_**1**_**-G** and **PN**_**3**_**-G** are expected to facilitate charge transfer form surface adsorbed molecules. The results indicate that doping N atoms in **PC**_**3**_**-G** offers opportunity to tune the properties of the surface. Next, we also conducted the molecular dynamics simulations (MD) to confirm the stability of the binding of P and N atoms on graphene surface by evaluating the thermodynamic stability of **PN**_**x**_**C**_**y**_**-G** at 300 K. The total simulation time of 2.0 ps is divided into 2500 steps in the NVT ensemble. The system energy and several random structures in the trajectory are presented in supplementary Fig. 3. The calculated results indicate that the single vacancy P doped graphene of **PC**_**3**_**-G** is shifted downward from the surface. As a result, the structure is deformed. However, no such severe deformation is seen in the substrate of **PC**_**3**_**-G**. For **PN**_**1**_**C**_**2**_**-G** and **PN**_**2**_**C**_**1**_**-G**, the structures are slightly deformed, and no atom is extrused from the substrate, which means that both surfaces remain stable at 300 K without atoms leaving the substrate. Obviously, the **PN**_**3**_**-G** bond length shows large fluctuations. The P and N bond distances increase during steps 50–90, and P and N bonds form at 100–250 steps. Thus, it is worth noting that the three coordinating N-atoms are not stable. The structures of P binding with neighboring N atoms are stable in **PN**_**1**_**C**_**2**_**-G** and **PN**_**2**_**C**_**1**_**-G** for the catalysis.

### Adsorption of reaction species on PN_x_C_y_-G

**Figure 3:**
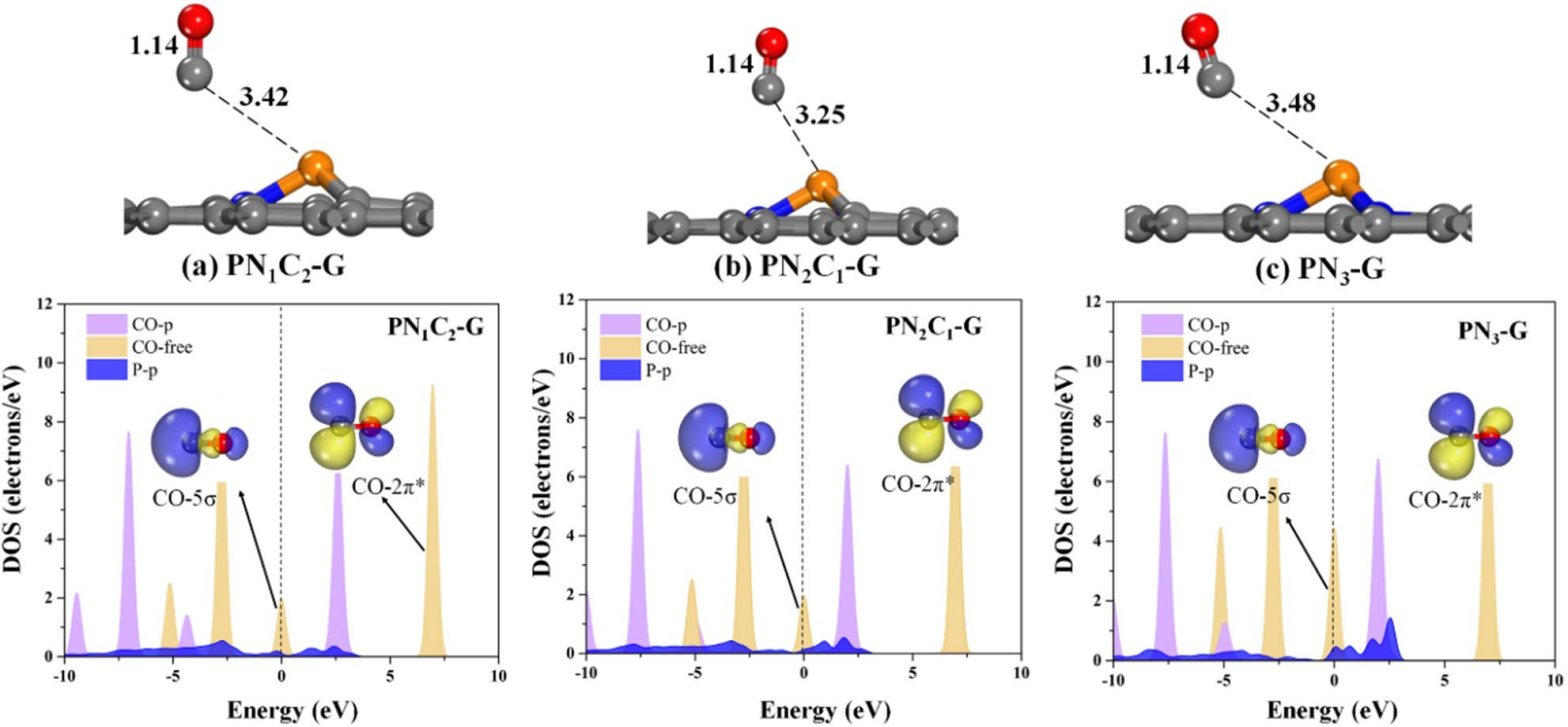
Optimized geometries and corresponding PDOS plots for the adsorption of CO over (**a**) **PN**_**1**_**C**_**2**_**-G**, (**b**) **PN**_**2**_**C**_**1**_**-G**, and (**c**) **PN**_**3**_**-G**. All bond distances are in Å. In the PDOS plots, the dashed line indicates the Fermi level (E_F_) level, which is set to zero.

**Figure 4:**
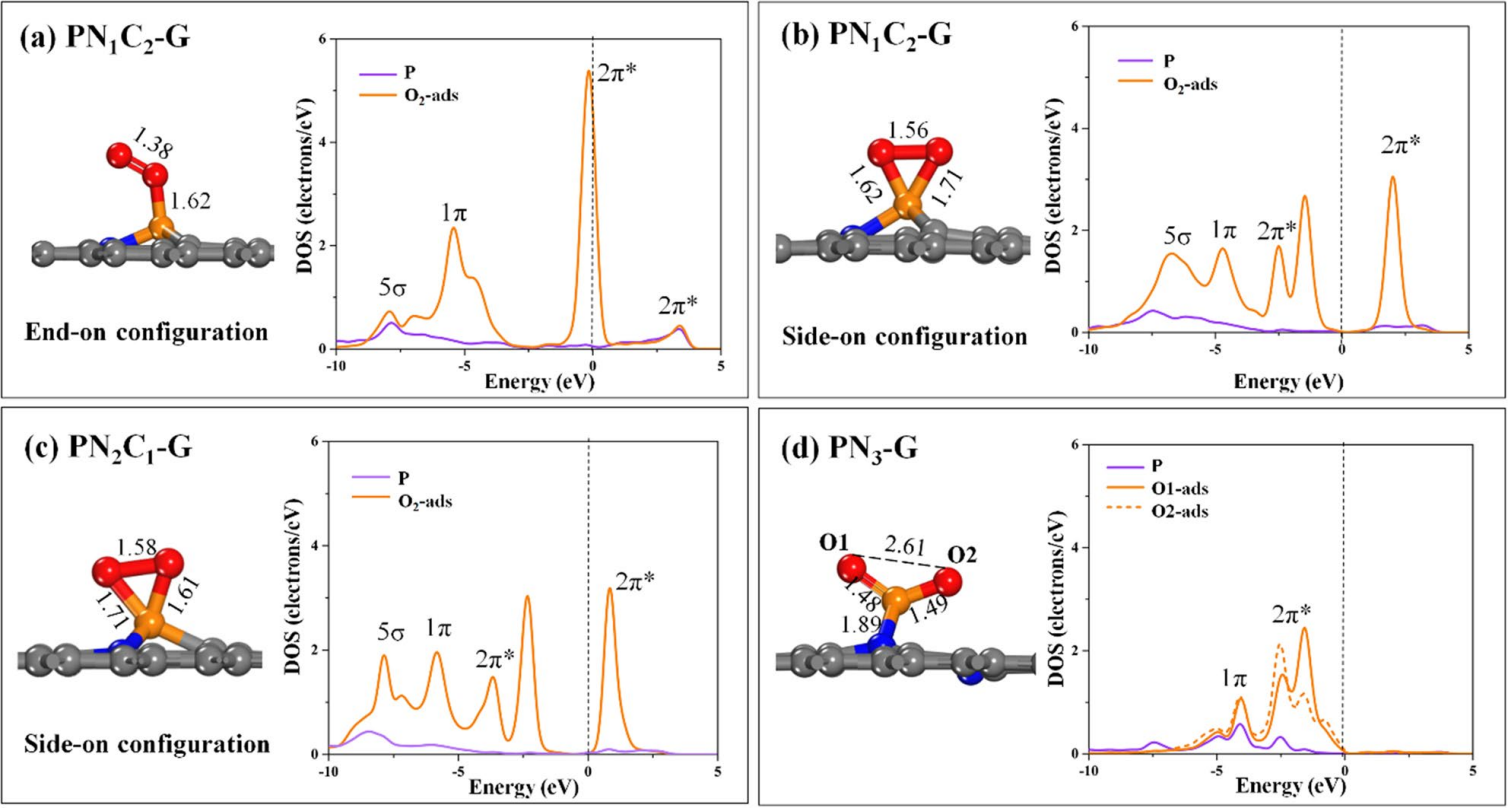
Optimized geometries and corresponding PDOS plots for the adsorption of O_2_ on (**a**) **PN**_**1**_**C**_**2**_**-G** (End-on), (**b**) **PN**_**1**_**C**_**2**_**-G**-(Side-on), (**c**) **PN**_**2**_**C**_**1**_**-G**, and (**d**) **PN**_**3**_**-G**. All bond distances are in Å. In the PDOS plots, the dashed line indicates the Fermi level (E_F_) level, which is set to zero.

The ability of the **PN**_**x**_**C**_**y**_**-G** graphene sheets to capture O_2_ and CO molecules around the active site was studied, which is an essential criterion to explore the catalytic activity towards the oxidation of CO. We begin with the understanding of the adsorption performance of the surface by analysing the adsorption energy and charge information of the gases on the surface which are summarized in supplementary Table 2. The structures of CO and O_2_ adsorbed on the surface are shown in Figs. 3 and 4. We now present the calculated results of the P and N co-dopants. We do not investigate the catalytic activity with those on single P-doped graphene (**PC**_**3**_**-G**). Our calculations find that the CO molecule bind through physisorption over the **PN**_**1**_**C**_**2**_**-G, PN**_**2**_**C**_**1**_**-G**, and **PN**_**3**_**-G** sheets, with adsorption energy (E_ads_) of 0.48, −0.20, and −0.23 eV, respectively. The results indicate that the C–O bond length remains unchanged with respect to the isolated state (1.14 Å) in the adsorbed configurations. Additionally, a small charge transfer from CO to the surface is negligible, which lies in the range of 0.001–0.008 |e|, clearly indicating that physisorption occurs between CO and the surface. Moreover, the plot of PDOS also confirms weak orbital overlap between the surface and the CO molecule around the Fermi level.

Next, the adsorption of O_2_ molecules on the surface is shown in an optimized geometry. Calculations indicate that the most favored adsorption of O_2_ molecules are chemisorbed over **PN**_**x**_**C**_**y**_**-G** sheets. Two configurations of O_2_ molecule adsorption over the **PN**_**1**_**C**_**2**_**-G** sheet exist are found, being the end-on configuration and the side-on configuration of O_2_**.** The end-on configuration (Fig. 4a) shows formation of an O–P chemical bond of 1.62 Å and the O–O bond of approximately 1.38 Å. As illustrated in Fig. 4a (right), the PDOS with the corresponding molecular orbital labels of the end-on configuration reveals that the hybridization of a P–p orbital and O_2_−2π* orbital slightly overlap above the Fermi level. Figure 4b illustrates the side-on configuration in which O_2_ molecules are nearly parallel to the surface. The O–O bond distance elongates to 1.56 Å from 1.24 Å in the isolated O_2_ molecule, which suggests an effective weakening of O–O bond. The E_ads_ for the end-on configuration is −0.54 eV, which is less than that of the side-on configuration of −1.76 eV, over **PN**_**1**_**C**_**2**_**-G**. Thus, the side-on configuration exhibits strong adsorption energy due to the O–O bond is parallel to the graphene surface and forms two chemical bonds with the P atom. Furthermore, PDOS corresponding molecular orbitals are plotted to show the strong adsorption of O_2_ (Fig. 4b, right). Strong hybridization occurs for the P atom and the side-on configuration of O_2_ on **PN**_**1**_**C**_**2**_**-G**, which may result in the ready decomposition of O_2_. Charge transfer is approximately 0.685 |e| from the P atom to the 2π* states of O_2_, which leads to a broadening and splitting of the 2π* states, and the elongation of the O–O bond to 1.56 Å. For **PN**_**2**_**C**_**1**_**-G**, the results show that O_2_ strongly adsorbs with side-on configuration (Fig. 4c) with an adsorption energy of −2.83 eV. The corresponding O–O binding distance is 1.58 Å, and there is a significant charge transfer from the surface to the O_2_ molecule, which causes a sizable elongation of the O–O distance. The PDOS in Fig. 4c (right) displays the strong P–p orbital and O_2_−2π* orbital hybridization. By contrast, we found that O_2_ molecules over **PN**_**3**_**-G** dissociate with about 2.61 Å elongation of the O–O distance. The elongation of O–O results in the P–N bond-breaking apart by about 2.75 Å. The adsorption energy is remarkably enhanced to −4.58 eV. As the result of removing a repulsive interaction from the negatively charged O atoms. The adsorption energy and charge transfer values increase with N coordination to the P atom. Additionally, the adsorption of O_2_ molecules over **PN**_**3**_**-G** destroys bond of P-N because the positive charge on the P atom is too large, indicating a significant tendency of this site to break incoming O_2_ molecules. Therefore, by increasing the coordination number of N atoms, the positive charge on the P increases, this phenomenon that does not benefit the adsorption of electron-rich molecules. Based on results indicate that the proper coordination of two instead of three N atoms around the P atom on the surface should benefit O_2_ adsorption. Next, we also investigated the electron density difference of **PN**_**x**_**C**_**y**_**-G** after the O_2_ molecules are adsorbed (supplementary Fig. 5). The results indicate an electron transfer from the surface to the adsorbed O_2_ molecule. The light blue and yellow in the electron density diagram demonstrate the capture and release of electrons, respectively. The results indicate that more electrons accumulate in the vicinity of the O_2_–P interface, while fewer electrons are located on the graphene surface. Therefore, increasing N coordination to P can facilitate charge transfer from the surface onto incoming gas molecules and significantly improve graphene's catalytic activity.

### Possible mechanisms for CO oxidation

O_2_ molecule on **PN**_**x**_**C**_**y**_**-G** exhibits stronger adsorption than CO. Thus, as well-known possible reaction mechanisms for the oxidation of CO to CO_2_ over a **PN**_**x**_**C**_**y**_**-G** nanosheet are studied via the Langmuir–Hinshelwood (LH), EleyeRideal (ER), and New EleyeRideal (NER) reactions^29,30^. In the ER mechanism pathway, the O_2_ molecule first adsorbs on the catalytic surface, and the adsorbed O_2_ molecule is attacked by CO to form a CO_2_ molecule via a CO_3_ intermediate. For the LH mechanism, reaction will start by the co-adsorption of O_2_ and CO molecules form a peroxo-type OOCO intermediate, which then dissociates to form a CO_2_ molecule. Otherwise, the NER mechanism involves the co-adsorption of two CO molecules, first physisorbed over the pre-adsorbed O_2_ molecule. Next, the physisorption of two CO molecules is close the pre-adsorbed O_2_ to forms an OOCCOO intermediate. Finally, the OOCCOO dissociates to form two CO_2_ molecules. These mechanisms are investigated in detail to find the preferred reaction pathway for CO oxidation.

### The 1st oxidation of CO via ER mechanisms

**Figure 5:**
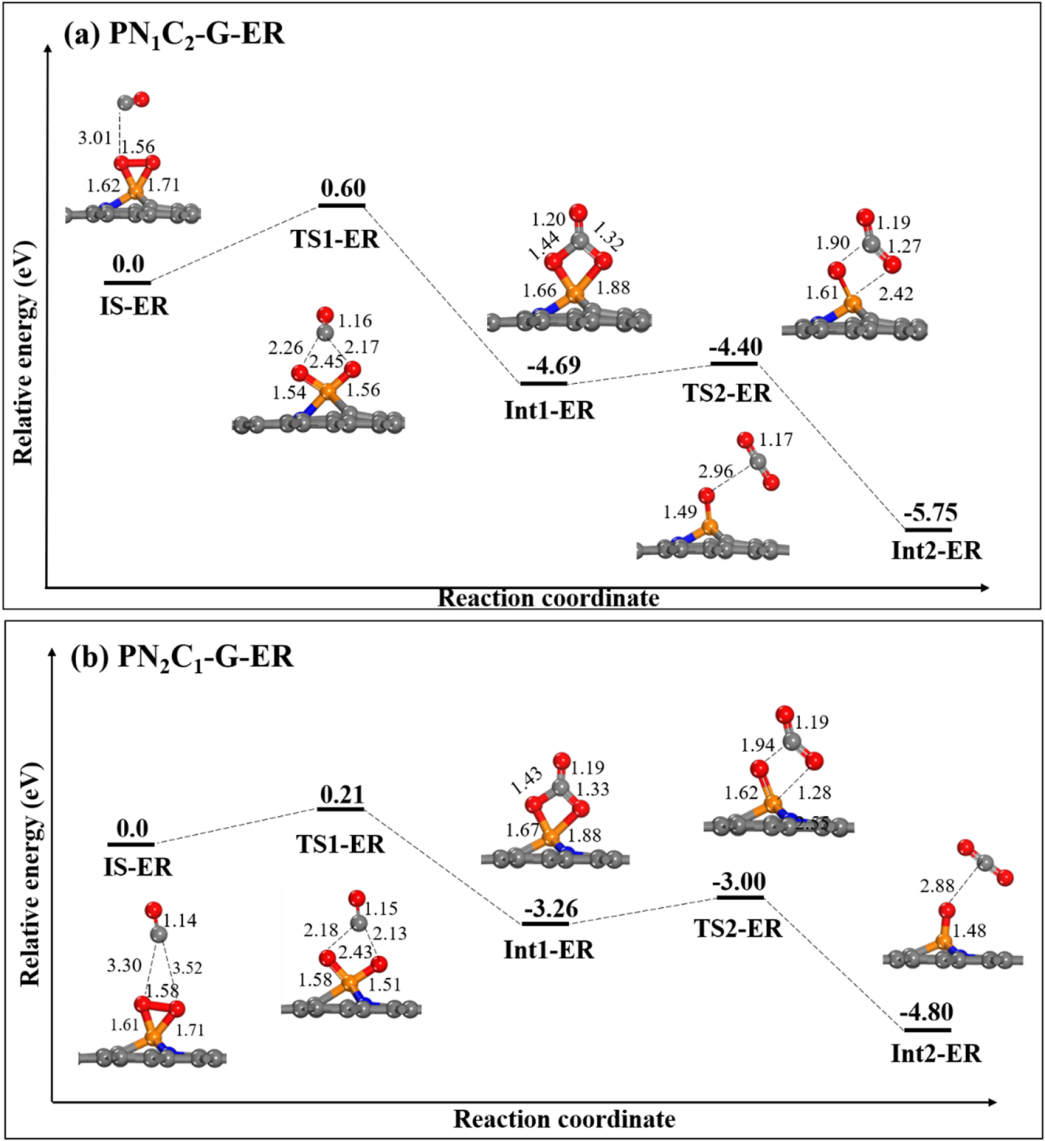
The potential energy surface diagram of CO oxidation via the first step in an ER mechanism (**a**) **PN**_**1**_**C**_**2**_**-G** and (**b**) **PN**_**2**_**C**_**1**_**-G**. All bond distances are in Å.

We first investigated the ER mechanism for CO oxidation. Figure 5a, b present the energy profiles for **PN**_**1**_**C**_**2**_**-G** and **PN**_**2**_**C**_**1**_**-G**; the initial structure for both surfaces is a side-on configuration. Unfortunately, we found the dissociation of the O_2_ molecule on **PN**_**3**_**-G**; therefore, we do not consider the CO oxidation via an ER mechanism on the **PN**_**3**_**-G.** Additionally, the end-on configuration of O_2_ on **PN**_**1**_**C**_**2**_**-G** as initial is ignored because when CO approaches the adsorbed O_2_, the O_2_ configuration changes from end-on to side-on, as shown in supplementary Fig. 7. Therefore, the only side-on configuration of O_2_ prefers to occur the CO oxidation via ER mechanism. For this mechanism to proceed, the CO is first close to the pre-adsorbed O_2_ on the P atom on the surface. In the physisorbed initial state (**IS-ER**), CO is 3.01 Å from the O_2_ in **PN**_**1**_**C**_**2**_**-G** and is 3.30 Å from **PN**_**2**_**C**_**1**_**-G**; the O–O bond distance remains unchanged. Next, CO attacks into the O–O bond and forms the CO_3_ structure as an intermediate (**Int1-ER**). The activation energy in this step is needed to form CO_3_ via the transition state (**TS1-ER**) for **PN**_**1**_**C**_**2**_**-G** and **PN**_**2**_**C**_**1**_**-G**, approximately 0.60 eV and 0.29 eV, respectively. Consequently, the **TS1-ER** for **PN**_**2**_**C**_**1**_**-G** is significantly more stable with stronger O–C–O bonds (2.18 and 2.13 Å) and O–P–O bonds (1.58 and 1.51 Å), see Fig. 5b. In the next step, the CO_3_ intermediate dissociates to form the first CO_2_ molecule via **TS2-ER** with an activation energy of 0.29 eV and 0.26 eV for **PN**_**1**_**C**_**2**_**-G** and **PN**_**2**_**C**_**1**_**-G**, respectively. Finally, the CO_2_ molecule desorbs, and one O atom still remains adsorbed to the P atom. Figure 5a, b indicate that the oxidation of CO via the ER mechanism is highly exothermic over **PN**_**1**_**C**_**2**_**-G** and **PN**_**2**_**C**_**1**_**-G.** We suggest that in the ER mechanism, CO oxidation on **PN**_**2**_**C**_**1**_**-G** is more favorable than **PN**_**1**_**C**_**2**_**-G**, according to the activation energy barriers.

### The 1st oxidation of CO via the LH mechanism

Now we shift our attention to CO oxidation via the LH mechanism. The most stable co-adsorption configuration of CO and O_2_ molecules in their initial states. We found that only **PN**_**1**_**C**_**2**_**-G** started with the co-adsorption of CO and O_2_ molecules on the surface(Supplementary Fig. 11). In its initial state (**IS-LH**), O_2_ is chemisorbed on the P atom and adopts a side-on configuration. CO is tilted over the surface and binding at the P atom with a bond distance of 3.33 Å. Note that the total adsorption energy of CO and O_2_ in this configuration is considerable about −3.09 eV. Moreover, the positive charge on phosphorus increases from 0.059 |e| in the side-on configuration of O_2_ on **PN**_**1**_**C**_**2**_**-G** to 1.342 |e| in the co-adsorbed configuration. Next, one of the P–O bond breaks, allowing CO to approach the P atom more closely, forming **TS1-LH**, providing the four-membered ring (OOCO) intermediate (**Int1-LH**) with an activation energy of 0.62 eV. Finally, the O–O and P–C bonds of **Int1-LH** lengthen by 1.83 Å and 2.62 Å, respectively. Then, CO_2_ molecule releases, leaving one O-atom remains adsorbed on the P atom at the surface via the transition state **TS2-LH** with the significant activation energy of 0.86 eV.

### The 2nd oxidation of CO via direct CO_2_ formation (pathway A)

After desorption of the first CO_2_, a single O* atom still remains bound to a P atom on the surface. We further investigated the second step of CO oxidation which is direct CO_2_ formation. In **pathway A** (Supplementary Fig. 12), the corresponding initial state **(IS-2A)**, the remaining O* atom interacts with an incoming CO molecule. First, the CO is physisorbed over the pre-adsorbed O* atom with an O*–CO binding distance of 3.03 Å for **PN**_**1**_**C**_**2**_**-G** and 3.12 Å for **PN**_**2**_**C**_**1**_**-G.** Then, CO approaches the O* atom to form CO_2_. The P–O bond distance is elongated to 1.86 Å for **PN**_**1**_**C**_**2**_**-G** and 1.85 Å for **PN**_**2**_**C**_**1**_**-G**. The results show that the residual O* is highly chemisorbed over the P atom, with adsorption energies of −4.86 eV (**PN**_**1**_**C**_**2**_**-G**) and −4.53 eV (**PN**_**2**_**C**_**1**_**-G**), resulting in high energy barriers of 0.71 eV (**PN**_**1**_**C**_**2**_**-G**) and 0.98 eV (**PN**_**2**_**C**_**1**_**-G**) to release the CO_2_ via transition state **TS1-2A**.

### The 2nd oxidation of CO via POCC-ring intermediate (pathway B)

**Figure 6:**
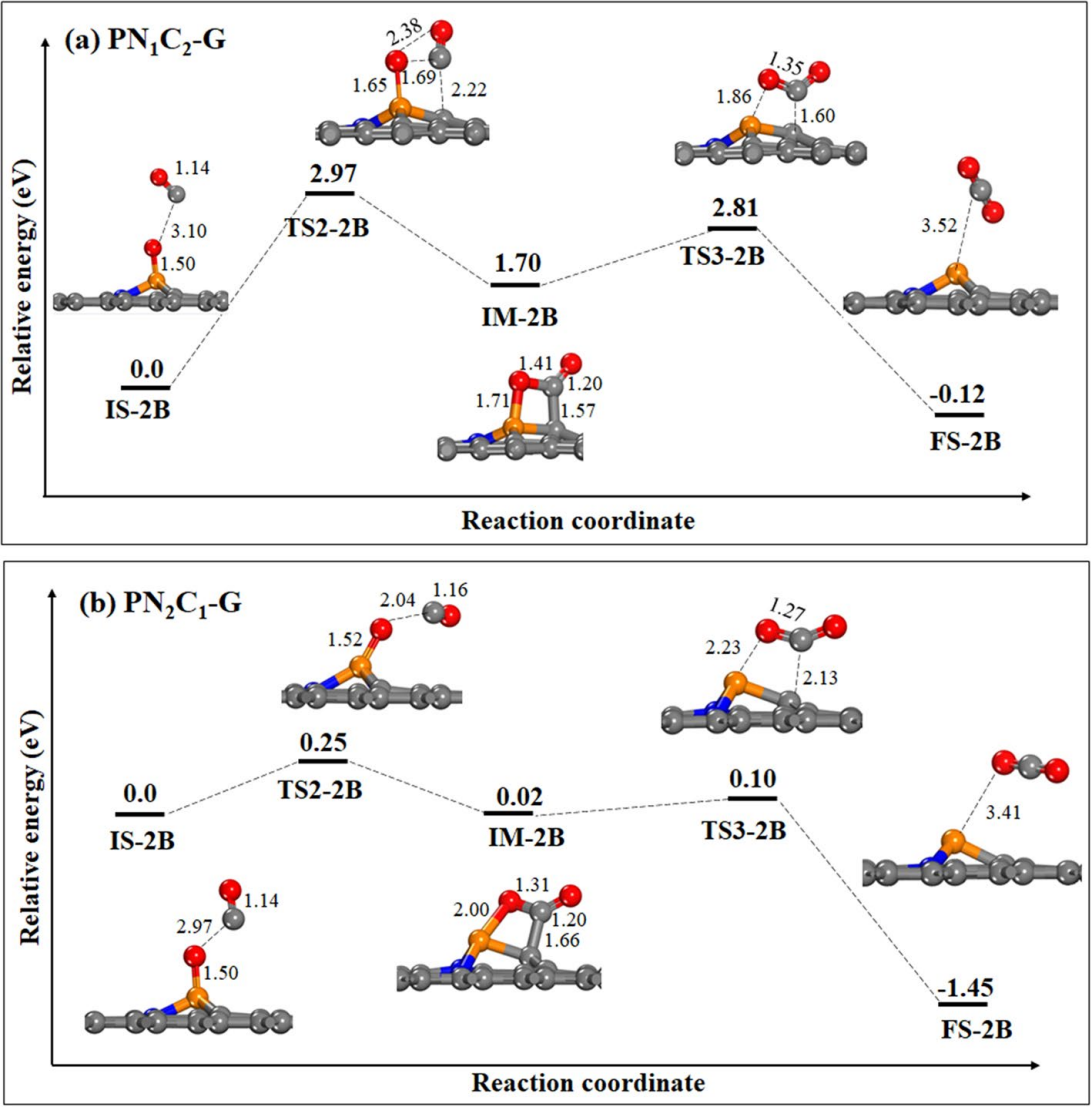
The potential energy surface diagram of CO oxidation via the second step in pathway B. (**a**) **PN**_**1**_**C**_**2**_**-G** and (**b**) **PN**_**2**_**C**_**1**_**-G**. All bond distances are in Å.

We investigated the second step of CO oxidation, CO_2_ formation via the POCC-ring intermediate as shown in **pathway B** (Fig. 6). For **PN**_**1**_**C**_**2**_**-G**, CO bonds to the surface at nearest C of P atom through C-bound, bringing two oxygen atoms close together and causing a strong repulsion which requires an energy barrier of 2.97 eV to form a POCC-ring intermediate. Surprisingly, when the N atom is added to the surface (**PN**_**2**_**C**_**1**_**-G**), CO is more likely to attach the remaining O* atom rather than the surface. As a result, two oxygen atoms are far apart with no repulsion, allowing the formation of the POCC-ring intermediate via **TS2-2B,** which has a significantly lower energy barrier of 0.25 eV. The second CO_2_ molecule is then released via **TS3-2B**, which has an activation energy of 1.11 eV for **PN**_**1**_**C**_**2**_**-G** and 0.08 eV for **PN**_**2**_**C**_**1**_**-G.** Furthermore, PDOS with the corresponding molecular orbital is plotted for the **IM-2B** intermediates in pathway B. The results show that the POCC-ring intermediate displays weak hybridization of the P-p orbital and 2π* orbital for **PN**_**2**_**C**_**1**_**-G**. This leads to small activation energy to break off the P–O bond on the **PN**_**2**_**C**_**2**_**-G** surface (Supplementary Fig. 13b). In addition, the reaction energy obtained for **PN**_**2**_**C**_**1**_**-G** is tiny at 0.08 eV, which clearly indicates the stability of the structure. Thus, a CO_2_ molecule forms and is quickly released from the surface at room temperature.

### The oxidation of CO via the NER mechanism

The energy profile of the NER mechanism involves simultaneous 1st and 2nd oxidation (Supplementary Fig. 14). In its initial state **(IS-NER**), two CO molecules are first physisorbed over the pre-adsorbed O_2_ molecule. The O–O bond distance of O_2_ lengthens until the five-membered ring intermediate **(Int1-NER)** forms via **TS1-NER** by overcoming the activation energies of about 0.50 eV and 0.34 eV for **PN**_**1**_**C**_**2**_**-G** and **PN**_**2**_**C**_**1**_**-G**, respectively. Next, the C–C bond of the five-membered ring intermediate breaks, leading to the formation of two CO_2_ molecules via **TS2-NER**. The dissociation of C–C bonds of **Int1-NER** is the rate-determining step that requires high activation energy of 1.46 eV for **PN**_**1**_**C**_**2**_**-G** and 1.19 eV for **PN**_**2**_**C**_**1**_**-G.**

### Rate constants

**Figure 7:**
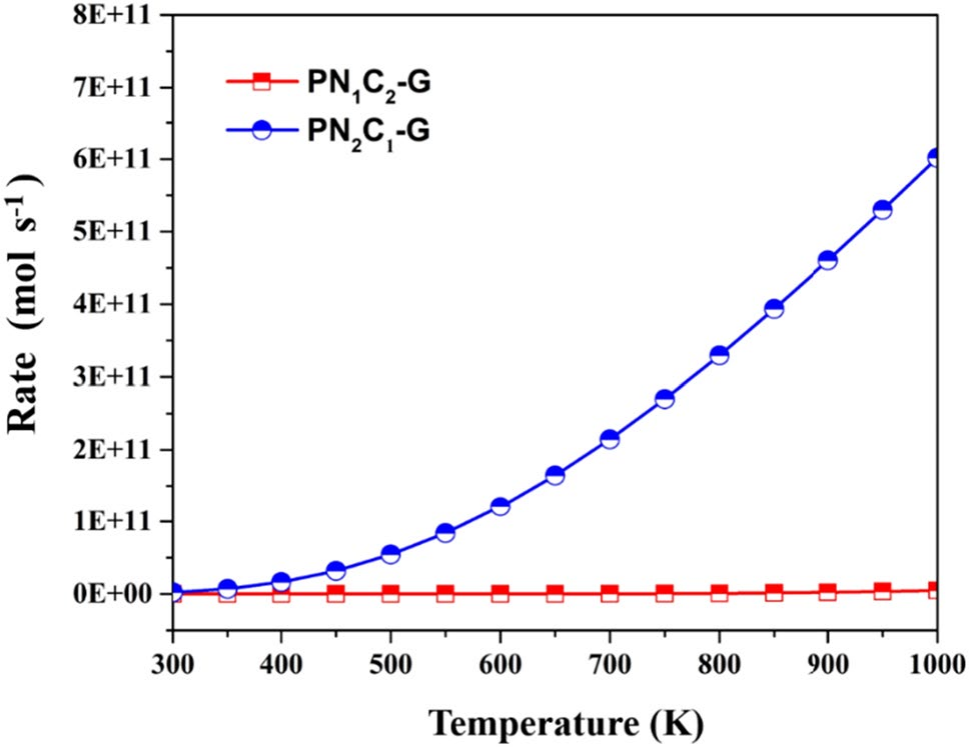
Rate of reaction for ER mechanism on PN_1_C_2_-G and PN_2_C_1_-G surface as function of temperature.

**Figure 8:**
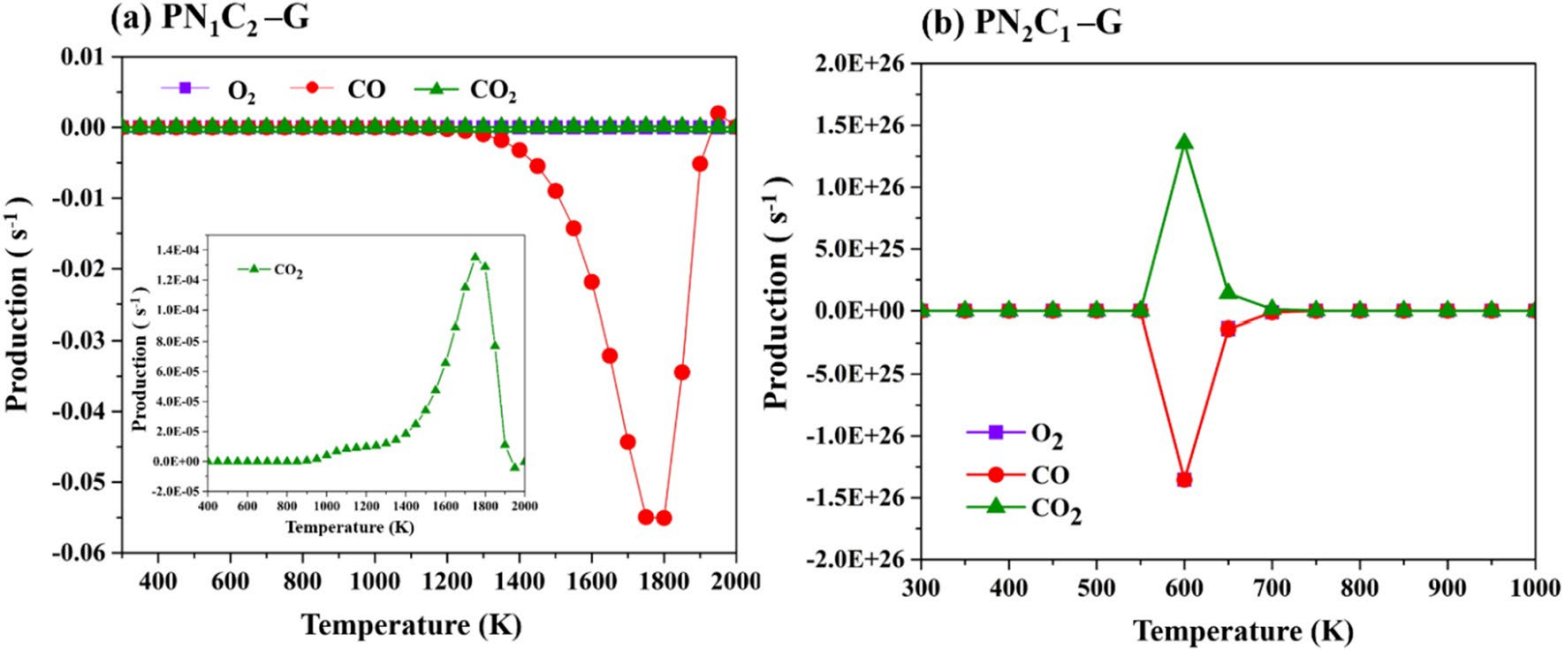
O_2_, CO, and CO_2_ production as a function of temperature during CO oxidation via ER mechanisms on (**a**) **PN**_**1**_**C**_**2**_**-G** and (**b**) **PN**_**2**_**C**_**1**_**-G** surface.

Our calculations found that CO oxidation reaction is catalyzed by two coordinated N atoms and single vacancy P-embedded graphene via ER mechanism, involving small activation energy (0.26 eV). Importantly, the catalytic model is used to obtain the rate constants of reaction. Thus, calculating the reaction rate of reaction are carried out by microkinetic simulation. The reaction rate for CO oxidation reaction on **PN**_**1**_**C**_**2**_**-G** and **PN**_**2**_**C**_**1**_**-G** surface as function of temperature are present in Fig. 7. The results show that ER mechanism on **PN**_**2**_**C**_**1**_**-G** surface displays much greater activity compared to **PN**_**1**_**C**_**2**_**-G** surface. The initial temperature of the reaction on **PN**_**2**_**C**_**1**_**-G** surface is about 350–400 K, which will start the reaction. Therefore, the two coordinated N atoms on single vacancy P-embedded graphene of **PN**_**2**_**C**_**1**_**-G** are more likely to occur at room temperature via ER mechanism, we found that temperature of the reaction increases with an increase of reaction rate for CO oxidation reaction. Additionally, the production rate of CO_2_ on both surface as function of temperature is present in Fig. 8. The ER mechanism on **PN**_**2**_**C**_**1**_**-G** surface exhibits a great production at maximum rate with the optimum temperature of 600 K (Fig. 8b), whereas the optimum temperature of **PN**_**1**_**C**_**2**_**-G** surface is dramatically increase to 1750 K (Fig. 8a). These results correspond to the rate determining step of overall reaction on **PN**_**1**_**C**_**2**_**-G and PN**_**2**_**C**_**1**_**-G** surfaces which are calculated to be 0.26 eV and 2.97 eV, respectively indicating that CO oxidation is remarkably favorable on **PN**_**2**_**C1-G** and hardly performed on **PN**_**1**_**C**_**1**_**-G** surface.

Moreover, we considered the O_2_ adsorption kinetic study. A plot of Gibbs free energy (G_ads_) value and temperature (T) of O_2_ reveal that G_ads_ decreases with an increase of T (Supplementary Fig. 15). The result predicts that the O_2_ molecule can adsorb easily in **PN**_**2**_**C**_**1**_**-G** at 370 K, while O_2_ molecule can adsorb on **PN**_**1**_**C**_**2**_**-G** and **PN**_**3**_**-G** at 450 K and 530 K, respectively. Thus, this result also supports that CO oxidation can occur in **PN**_**2**_**C**_**1**_**-G** at low temperatures.

### Catalytic performance of PN_x_C_y_-G

**PN**_**x**_**C**_**y**_**-G** sheets, where x and y = 1 and 2, exhibit excellent catalytic performance for CO oxidation due to the synergistic effect of P and N atoms on the graphene surface. In the investigation of adsorption of O_2_ and CO on P and N co-doped graphene, the P and N doping models exhibit appropriate activities to adsorb O_2_ and are catalysts for the oxidation of CO. Moreover, we found that the O_2_ molecule shows intense adsorption energy on the **PN**_**3**_**-G** surface. Therefore, P and three coordination N atoms are not beneficial for the adsorption of electron-rich molecules because of the additional positive charge on the P atom. Additionally, the adsorption of O_2_ on the **PN**_**3**_**-G** surface breaks the bond between P and N on the surface. Moreover, G_ads_ value of O_2_ associated T indicates that O_2_ molecule activates easily in **PN**_**2**_**C**_**1**_**-G** at 370 K, which is much lower than **PN**_**2**_**C**_**1**_**-G** and **PN**_**3**_**-G**. The above results reflect that the catalytic performance of the first and second steps for CO oxidation on the **PN**_**2**_**C**_**1**_**-G** surface via the ER mechanism involves activation energy of less than 0.50 eV. Hence, based on these results, we find that the ER mechanism should be more favorable than the LH and NER mechanisms for the oxidation of CO molecules over a **PN**_**2**_**C**_**1**_**-G** surface. We compared the activation energies of the rate-determining step for CO oxidation on **PN**_**x**_**C**_**y**_**-G** with those on a different metal-free graphene-based catalyst (Supplementary Table 3). We note that the calculated small activation energy for the ER mechanism on **PN**_**2**_**C**_**1**_**-G** is comparable with those over P-doped graphene. The results indicate that an increase in the catalytic activity of P and N co-doped graphene for CO oxidation is achievable by two-coordinated N atoms to the P atom on graphene.

## Conclusion

The effects of P and N co-doping and N dopant concentration on catalytic activity of graphitic **PN**_**x**_**C**_**y**_**-G**, where x and y = 0, 1, and 2, toward CO oxidation by O_2_ are investigated by DFT calculations. The results show that N doped single vacancy P-embedded graphene can considerably enhance the surface reactivity of graphene compared to P-doped and N-doped species. The calculated results of the surface properties are related to the large electronegativity difference among the P, N, and C atoms, which induces a positive charge on the P atom. Additionally, two and three coordinated N atoms doping exhibit the small energy gap between valence and conduction band due to the conduction band shifts down to Fermi level. Thus, increasing two or three coordinated N to P can facilitate charge transfer from the surface onto incoming gas molecules and greatly improve graphene's catalytic activity. However, three coordinated N atoms of **PN**_**3**_**-G** show a weak interaction, easily deformed and extrused from the substrate. On the other hand, the interaction between the O_2_ molecule and **PN**_**3**_**-G** is strong as shown in the calculated adsoption energy, suggesting that CO oxidation cannot be initiated over these surfaces. Moreover, Gibbs free energy (G_ads_) of O_2_ molecule adsorb on **PN**_**3**_**-G** surface at high temperature (530 K), while the temperature for adsorption of O_2_ molecule on **PN**_**1**_**C**_**2**_**-G** and **PN**_**2**_**C**_**1**_**-G** are 450 K and 370 K, respectively. The synergistic effect of P and two-coordinated N atoms effectively improve the catalytic activity for excellent catalytic performance in CO oxidation. **PN**_**2**_**C**_**1**_**-G** catalyzes CO via the ER mechanism at 0 K to overcome the very low energy barrier of 0.26 eV for the first and 0.25 eV for the second CO oxidation process, then releasing 2 molecules of CO_2_. The thermodynamic study reveals that CO oxidation can spontaneously take place on **PN**_**2**_**C**_**1**_**-G** at room temperature. The calculated rate-determining step via ER mechanism of **PN**_**2**_**C**_**1**_**-G** at 298.15 K is about 0.77 eV. The reaction rate at 298.15 K is calculated to be 5.36 × 10^16^ mol s^–1^. Our findings also indicate that for the CO oxidation reaction over **PN**_**1**_**C**_**2**_**-G** and **PN**_**2**_**C**_**1**_**-G**, the ER mechanism is more favorable than the LH and NER mechanism. Moreover, the calculated activation energy for the CO oxidation reaction through the ER mechanism is comparable or even smaller than those of metal-free-based catalysts. Therefore, N co-doped on single vacancy P-embedded graphene show effective catalytic for CO oxidation reaction. Our new finding provides guidelines for designing highly efficient metal-free catalysts.

## Supplementary Information

Supplementary Information.

## Data Availability

No datasets were generated or analysed during the current study.
