# Supplementary material for: Theoretical study of metal-free catalytic for catalyzing CO-oxidation with a synergistic effect on P and N co-doped graphene
Source: Sci Rep. 2022 Jun 21;12:10439. doi: 10.1038/s41598-022-14286-8 (PMC9213554; doi:10.1038/s41598-022-14286-8)
Supplement: Supplementary file 1 — Supplementary Information. [file 41598_2022_14286_MOESM1_ESM.docx]

**Supplementary Information**

**Theoretical study of metal-free catalytic for catalyzing CO-oxidation with a synergistic effect on P and N co-doped graphene**

Sarinya Hadsadee ^1^, Siriporn Jungsuttiwong ^2*^, Rui-Qin Zhang ^3^, Thanyada Rungrotmongkol ^1,4*^

^1^*Center of Excellence in Biocatalyst and Sustainable Biotechnology, Department of Biochemistry, Faculty of Science, Chulalongkorn University, Bangkok 10330, Thailand.*

^2^*Department of Chemistry and Center of Excellence for Innovation in Chemistry, Ubon Ratchathani University, Ubon Ratchathani 34190, Thailand E-mail:* [siriporn.j@ubu.ac.th](mailto:siriporn.j@ubu.ac.th)

^3^*Department of Physics and Materials science and Centre for Functional Photonics (CFP), City University of Hong Kong, Hong Kong, China.*

^4^*Program in Bioinformatics and Computational Biology, Graduate School, Chulalongkorn University, Bangkok 10330, Thailand. E-mail:* [thanyada.r@chula.ac.th](mailto:thanyada.r@chula.ac.th)

**
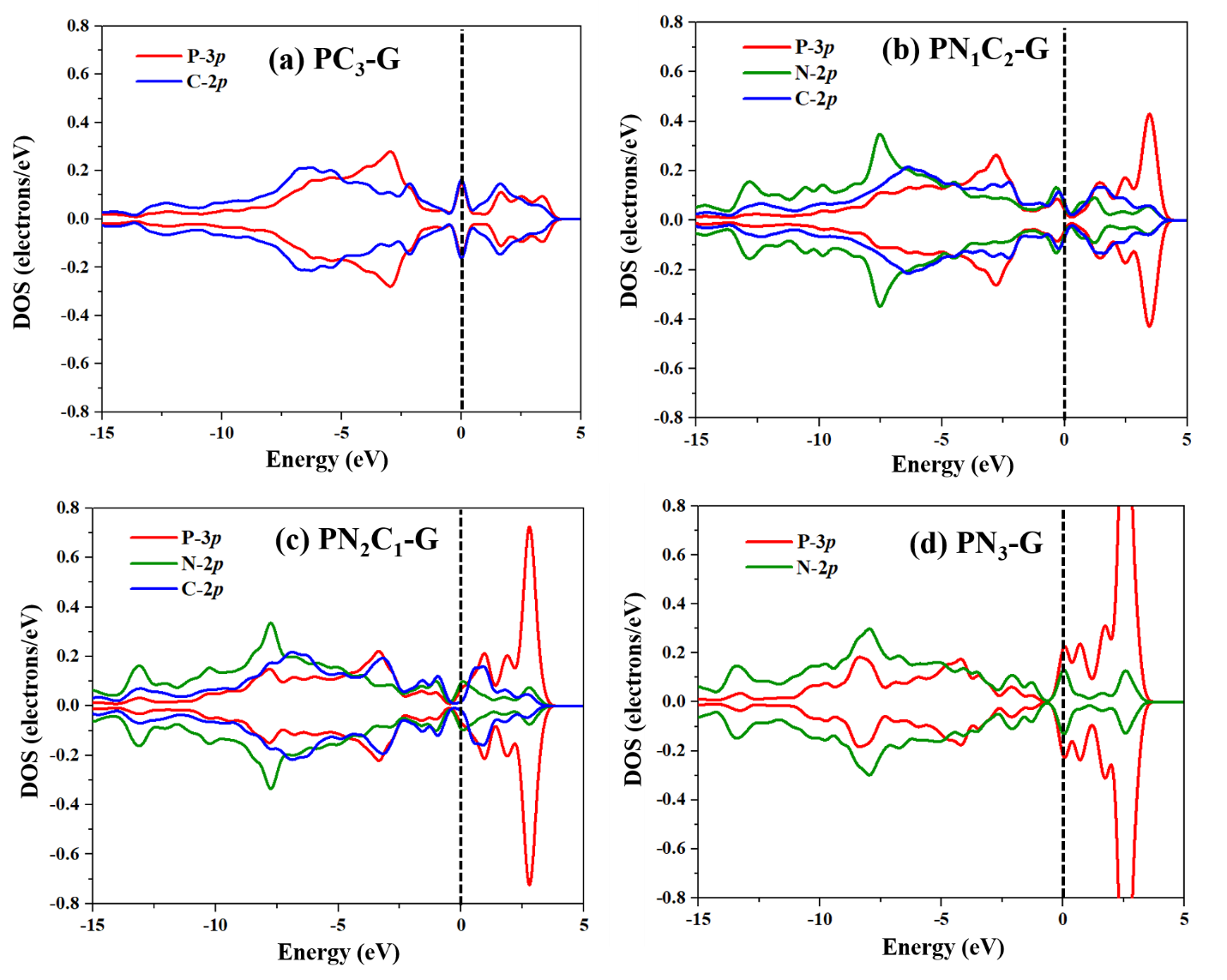
**

Figure 1. PDOS plots for (a) single vacancy P-embedded graphene, (b) N-doped single vacancy P-embedded graphene, (c) two N-doped single vacancy P-embedded graphene, (d) three N-doped single vacancy P-embedded graphene. The Fermi level is set to zero.


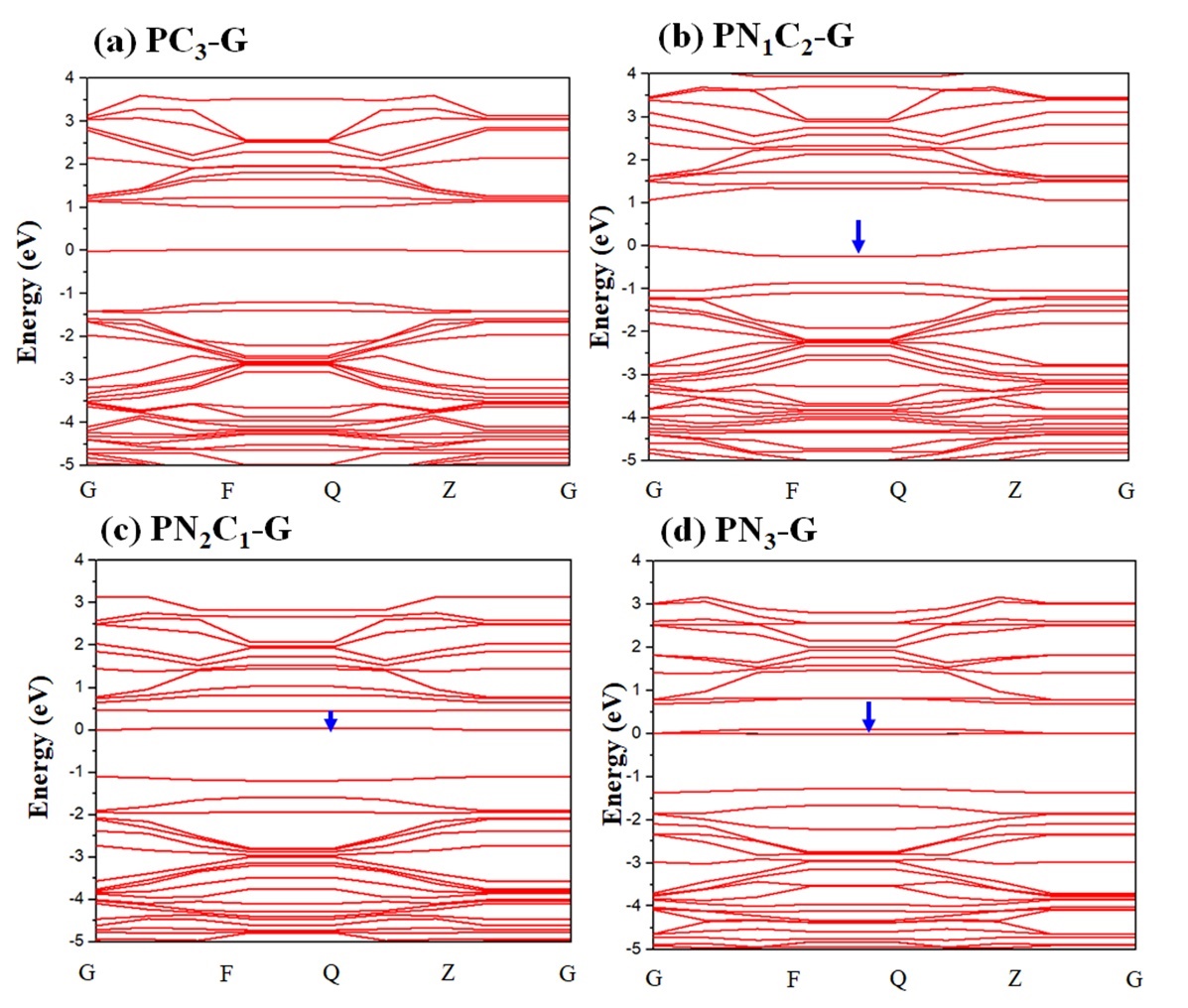


Figure 2. The band structures of (a) PC_3_-G, (b) PN_1_C_2_-G, (c) PN_2_C_1_-G, and (d) PN_3_-G monolayers, which show unpaired spins.


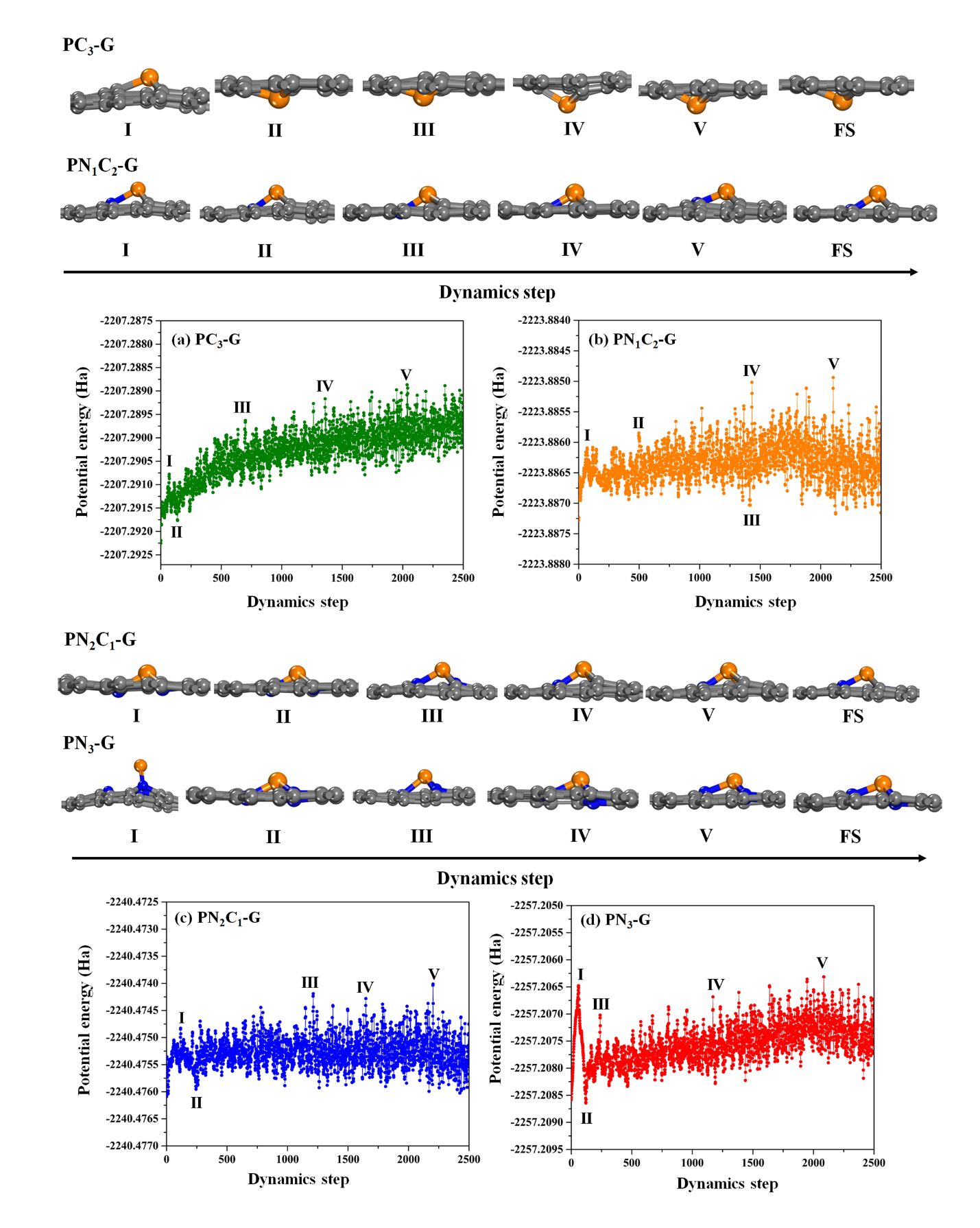


Figure 3. The potential energy as a function of time, obtained from molecular dynamics simulations (MD).


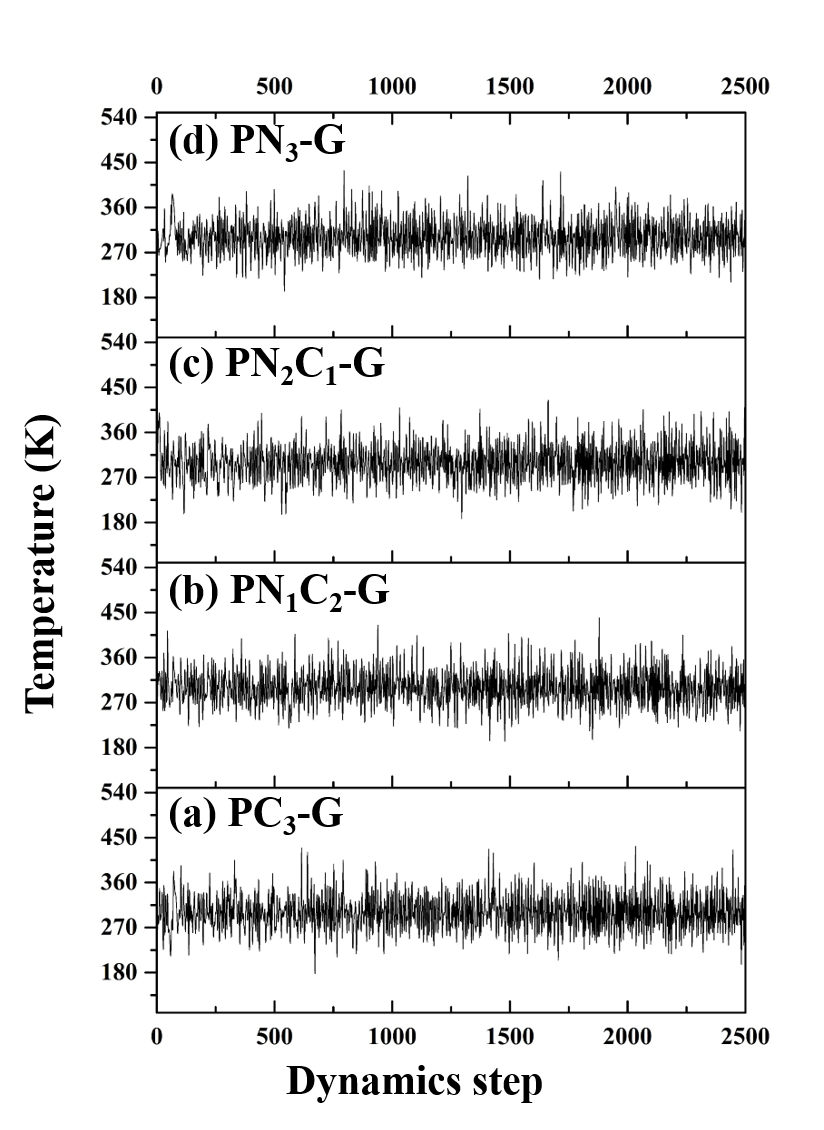


**Figure 4.** The temperature as the function of time obtained from molecular dynamic simulations (MD).

**
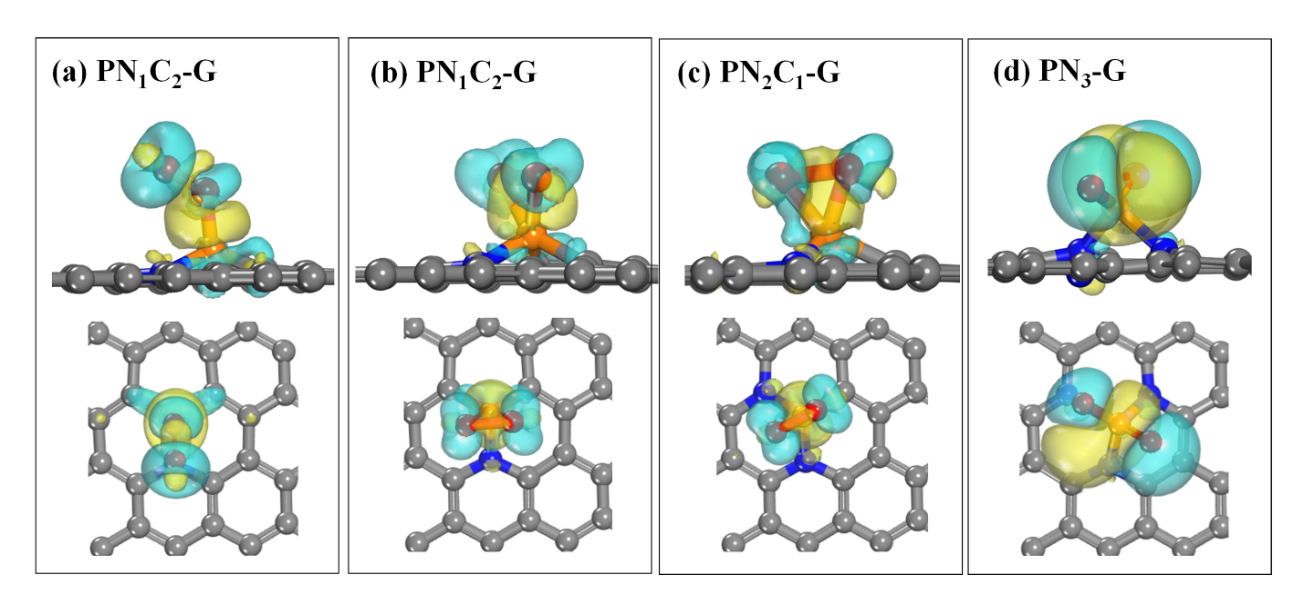
**

Figure 5. Electron density difference maps of O_2_ adsorption on (a) PN_1_C_2_-G-(End-on), (b) PN_1_C_2_-G-(Side-on), (c) PN_2_C_1_-G, and (d) PN_3_-G. (the isovalue is ±0.02)

**
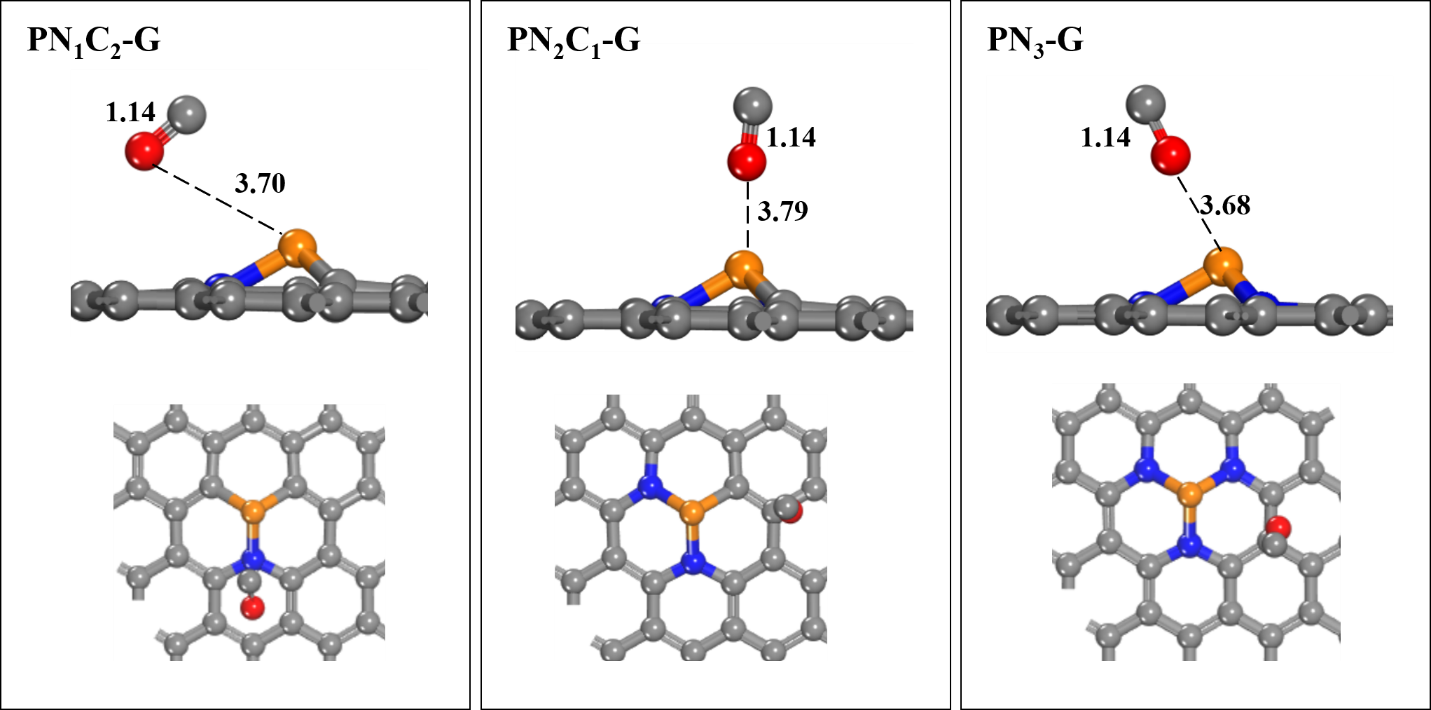
**

**Figure 6**. Optimized geometries CO molecule on PNC-G surfaces.

**
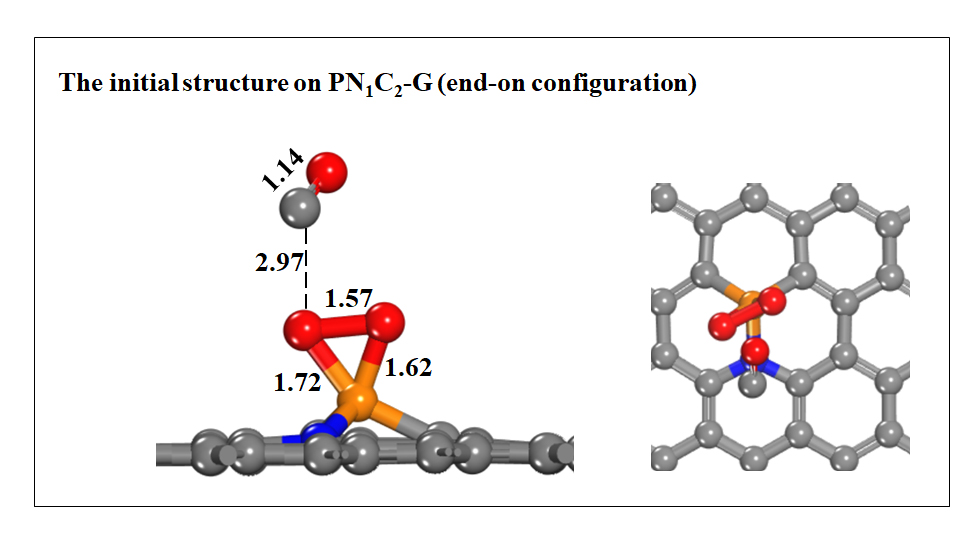
**

**Figure 7**. Optimized initial structure of O_2_ and CO molecules for end-on configuration of O_2_ on **PN_1_C_2_-G**

**
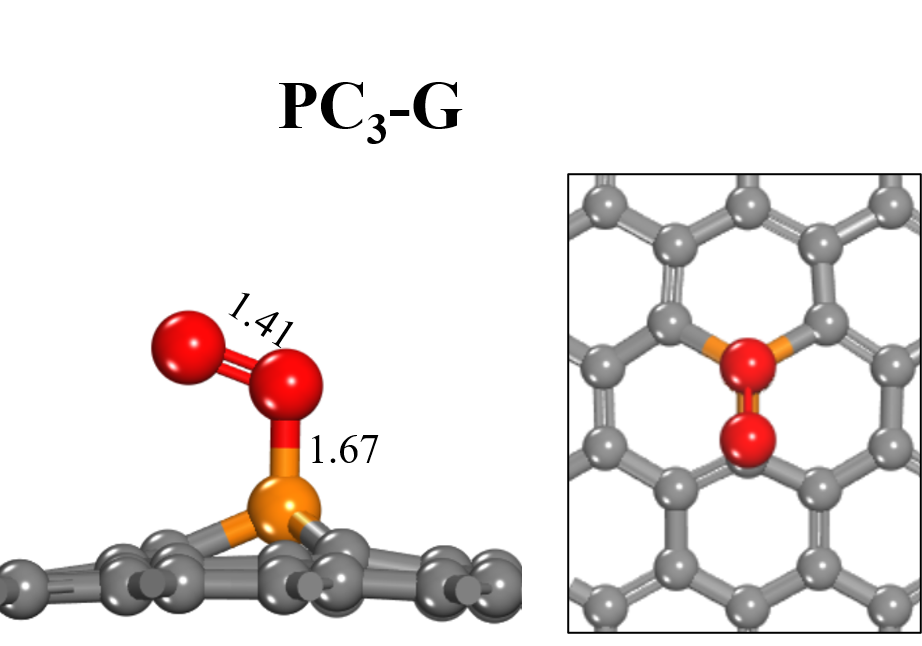
**

**Figure 8**. Optimized geometries O_2_ molecule on **PC_3_-G.**


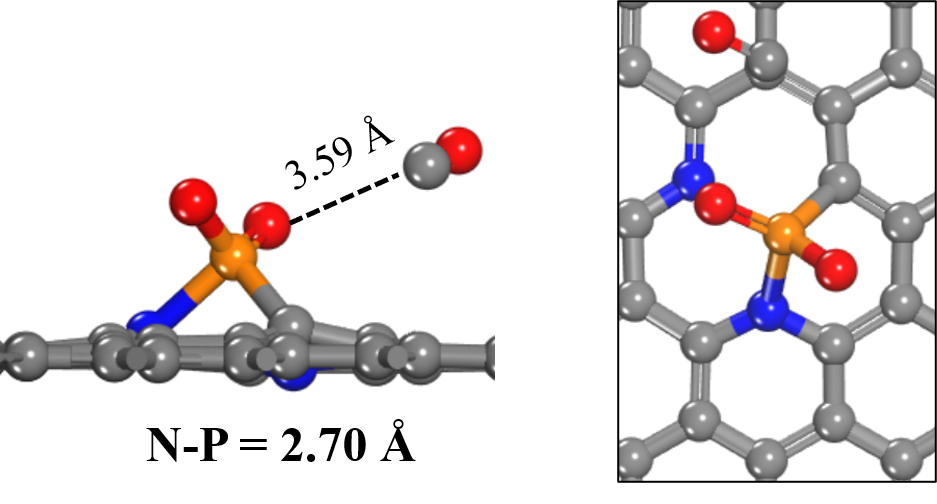


**Figure 9**. The co-adsorption of O_2_ and CO molecules on **PN_2_C_1_-G.**


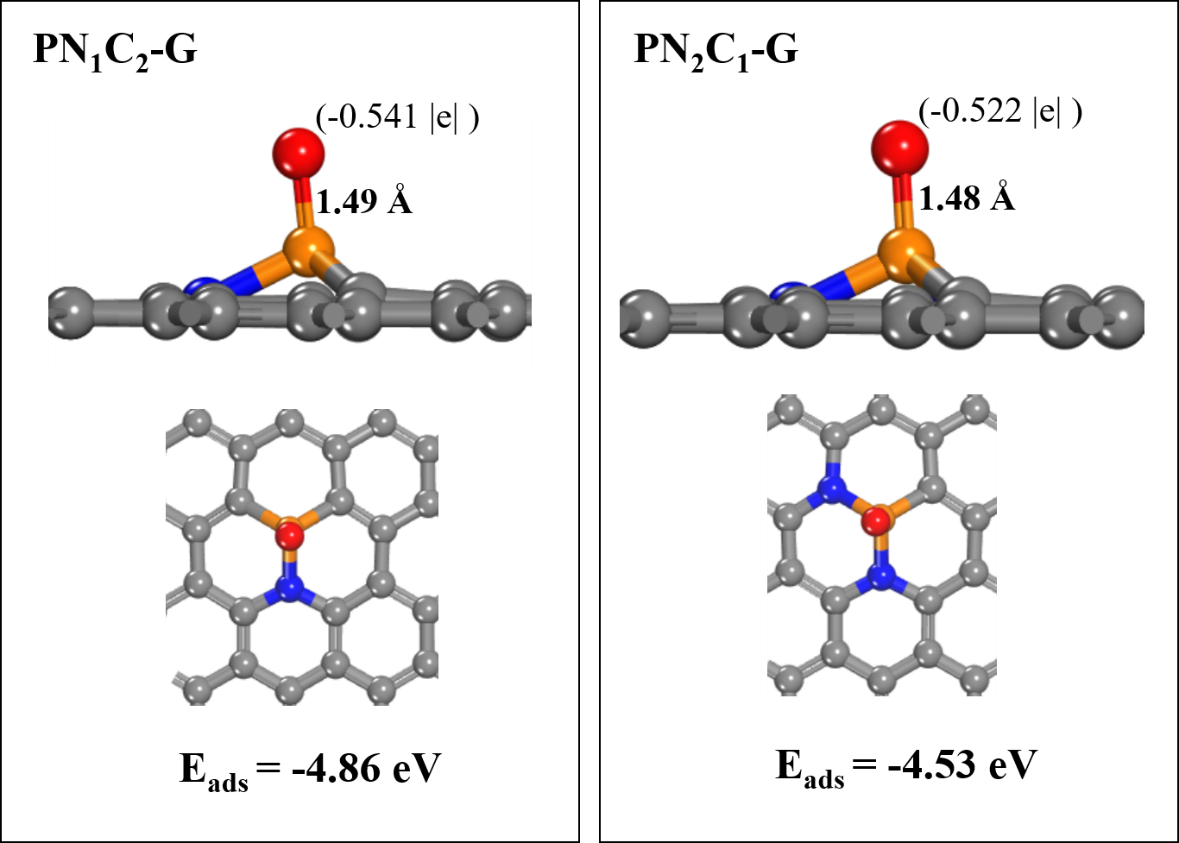


**Figure 10.** Optimized geometries for the adsorption of O on **PN_1_C_2_-G** and **PN_2_C_1_-G**

**
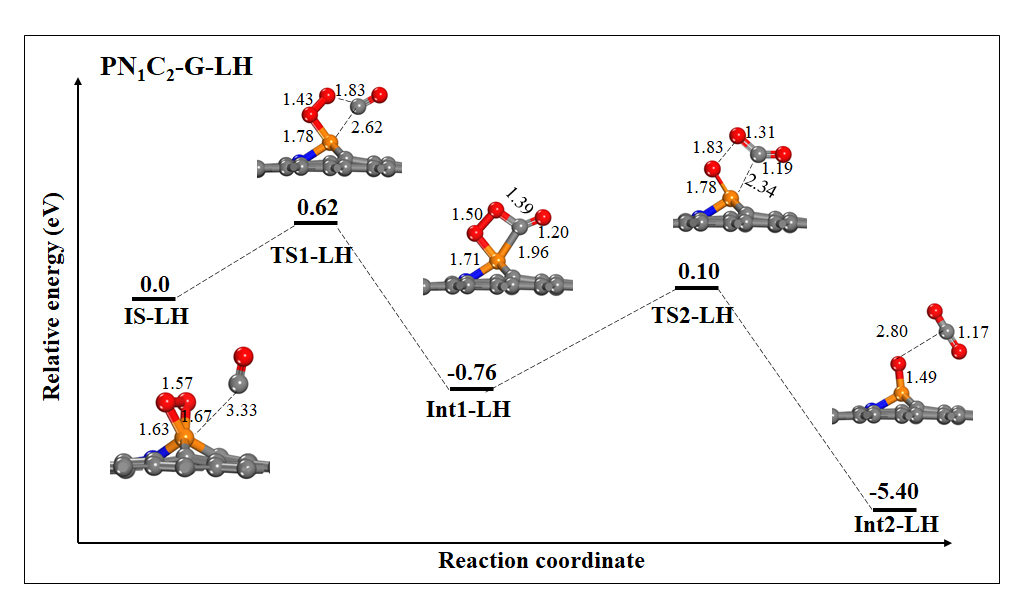
**

Figure 11 The potential energy surface diagram of CO oxidation shows the LH mechanism for PN_1_C_2_-G. All bond distances are in Å.


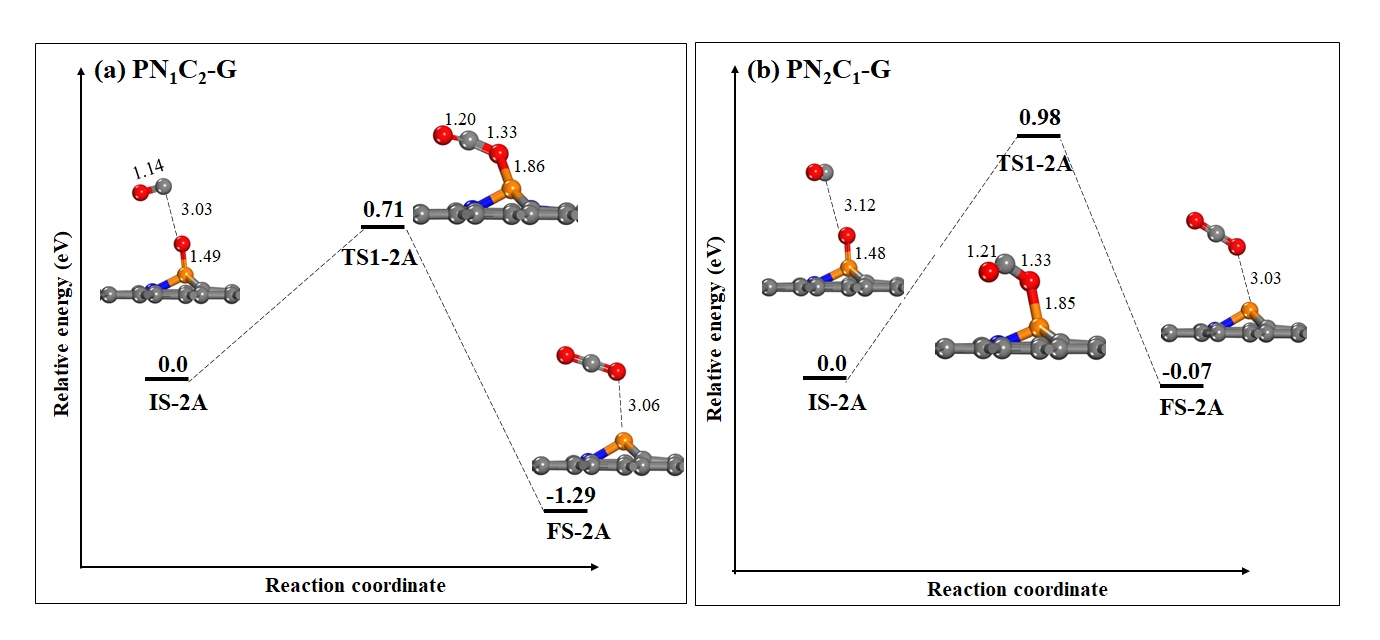


Figure 12. The potential energy surface diagram of CO oxidation via the second step in pathway A. (a) PN_1_C_2_‑G and (b) PN_2_C_1_-G. All bond distances are in Å.


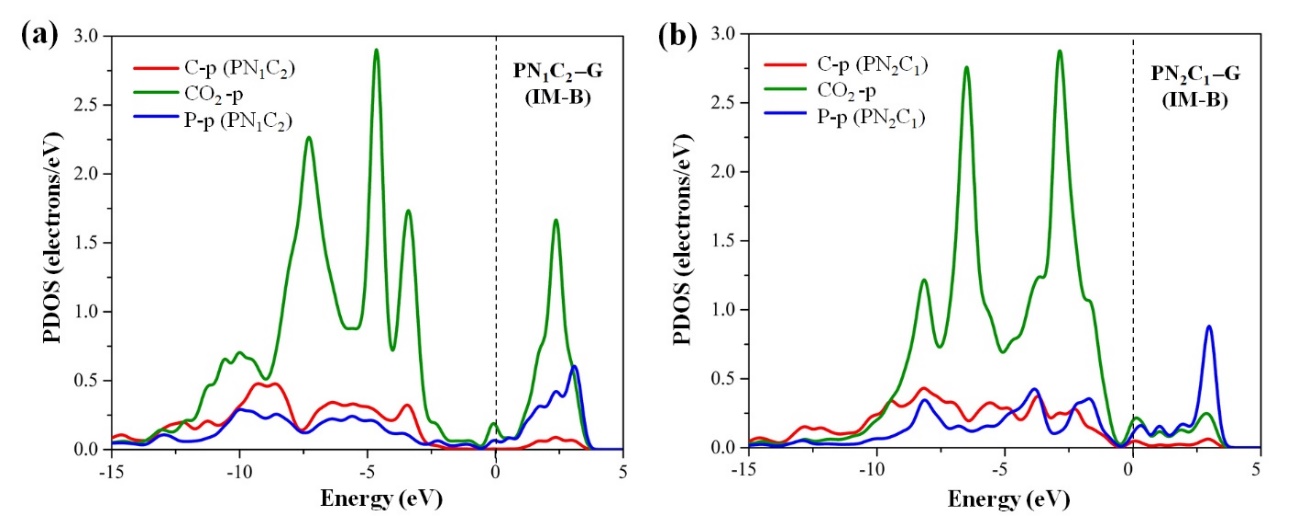


Figure 13. The corresponding PDOS plots for the intermediate (IM-B) of OCO on (a) PN_1_C_2_-G and (b) PN_2_C_1_-G in pathway B.


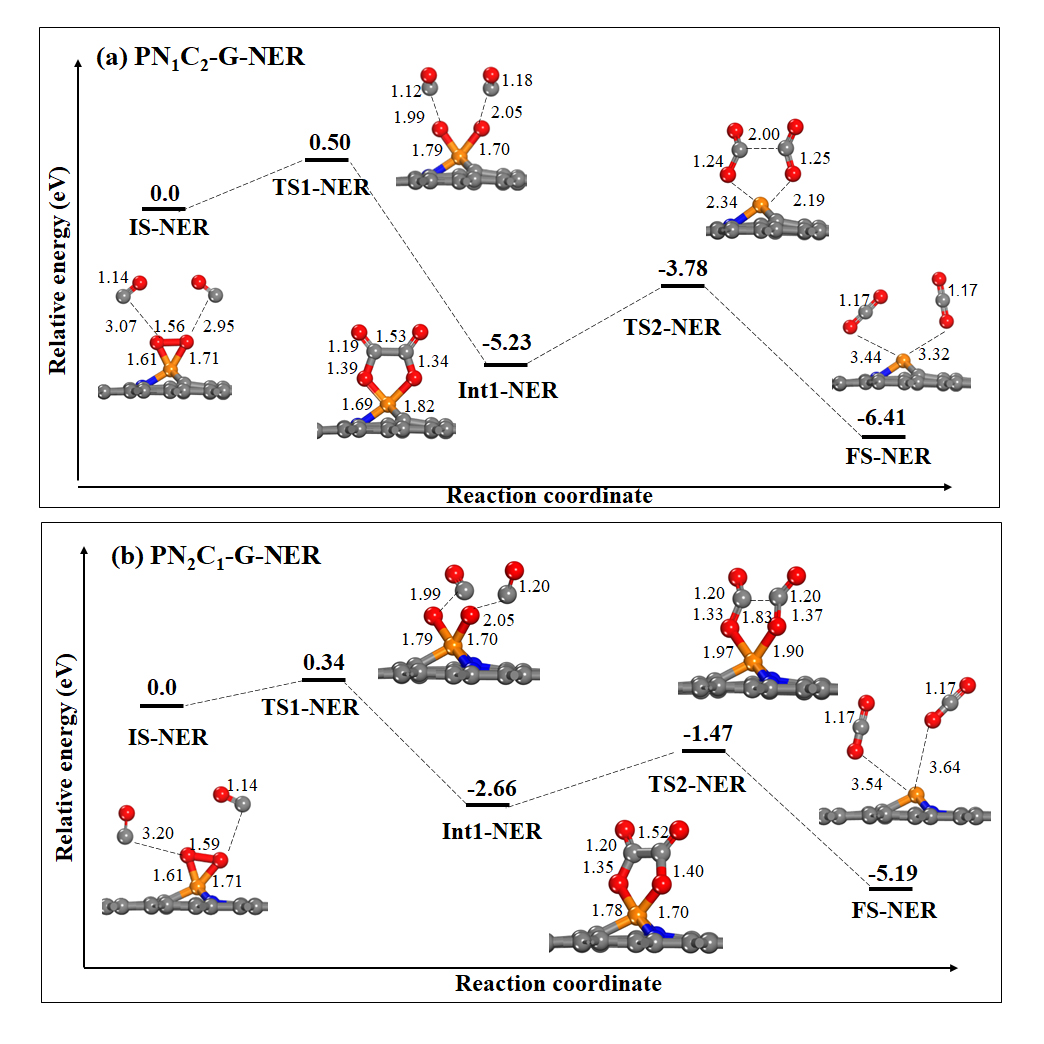


Figure 14. The potential energy surface diagram for CO oxidation via a NER mechanism (a) PN_1_C_2_-G and (b) PN_2_C_1_-G. All bond distances are in Å.


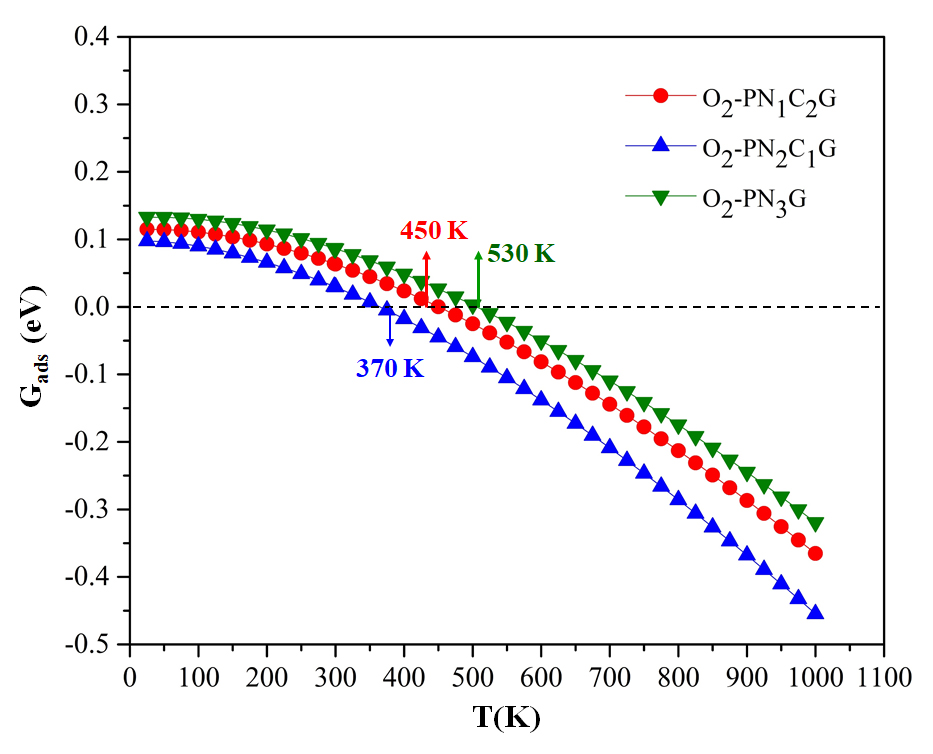


Figure 15. Gibbs free energy dependent on the temperature obtained from DFT calculations.

**
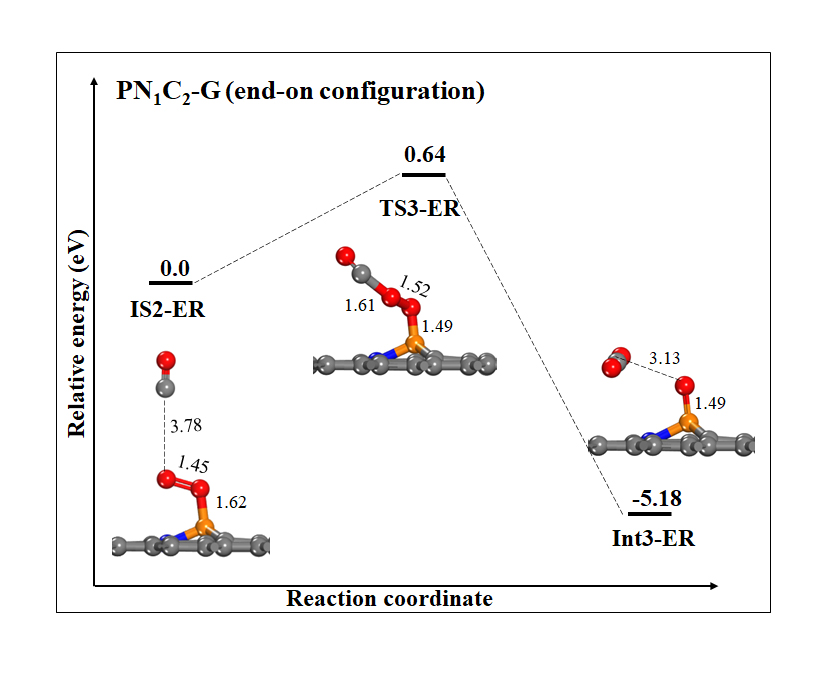
**

**Figure 16.** The potential energy surface diagram of CO oxidation via an ER mechanism the first step of PN_1_C_2_-G for end-on configuration of O_2_. All bond distances are in Å.

**
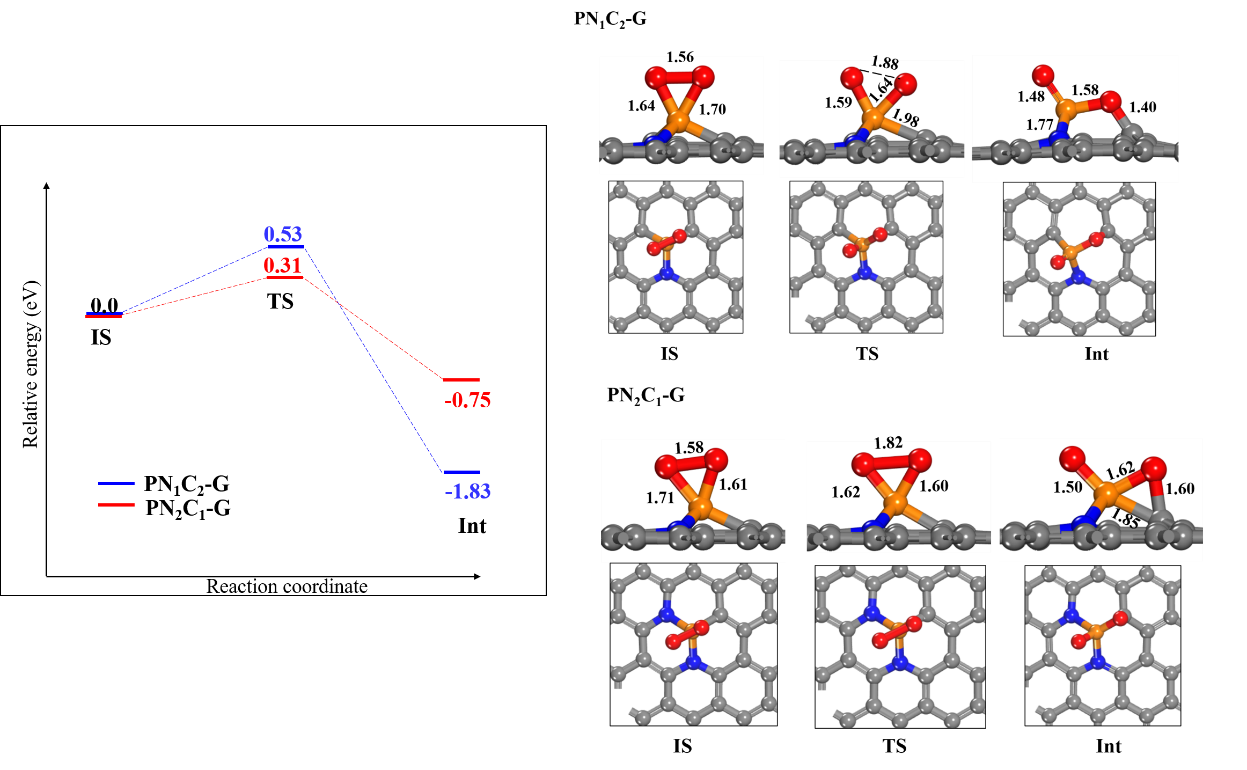
**

**Figure 17.** The energy path for O_2_ molecule dissociation (O_2_ → 2O, blue and red represent reactions that take place in PN_1_C_2_-G and PN_2_C_1_-G, respectively).


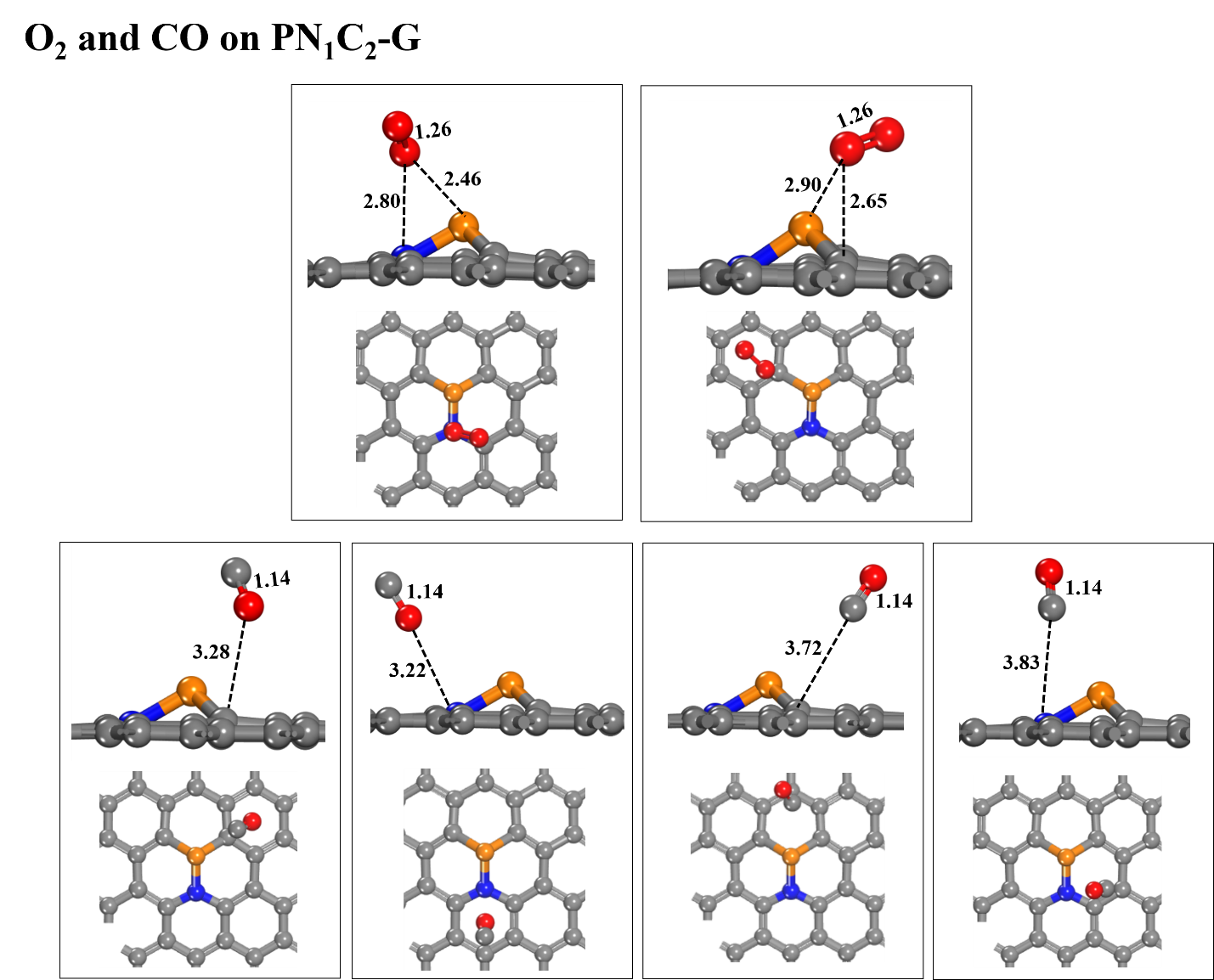


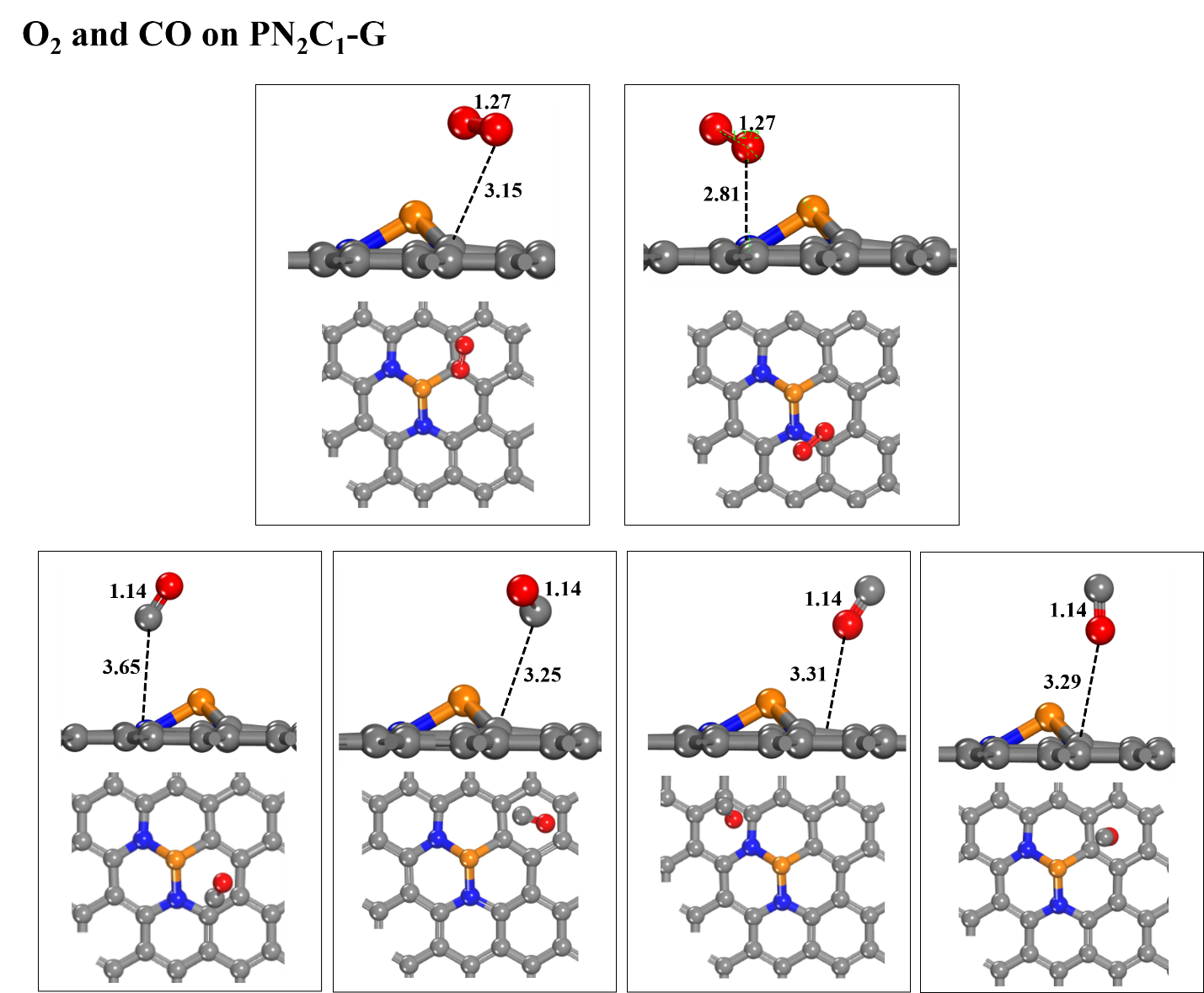


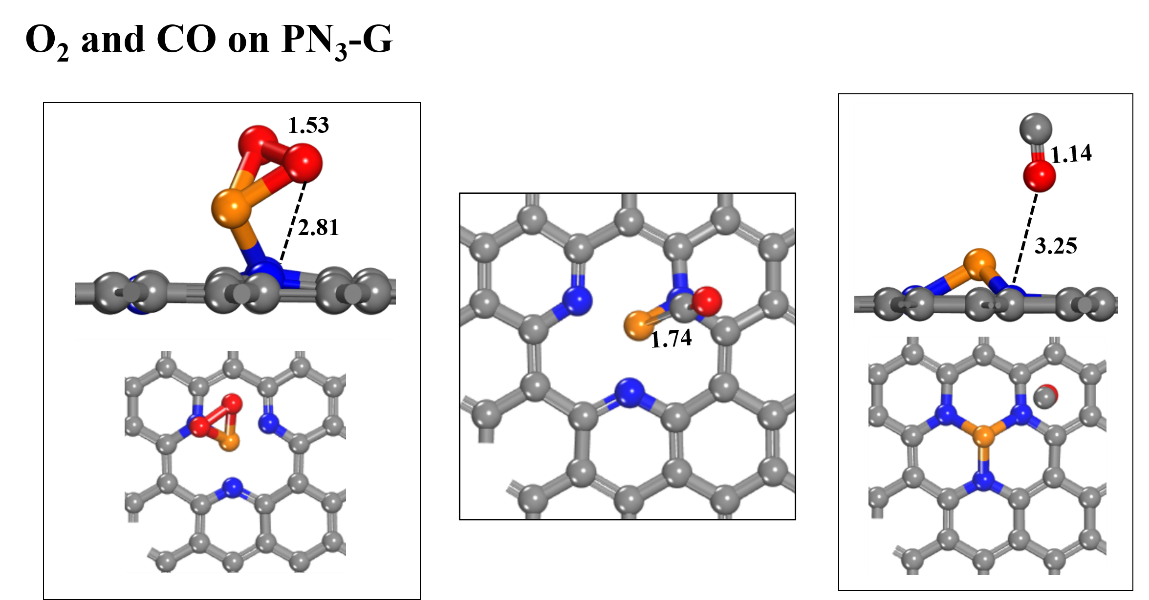


**Figure 18.** possible configuration adsorption for O_2_ and CO to find the possible reaction mechanisms.

**Table 1** Calculated atomic charges on the P atom (q_P_, |e|) and formation energies (E_form_, eV) of **PN_x_C_y_-G** graphitic monolayers.

| **Surface** | **q_P_** | **E_form_** |
| --- | --- | --- |
| PC_3_-G | 0.638 | -1.76 |
| PN_1_C_2_-G | 0.692 | -1.90 |
| PN_2_C_1_-G | 0.810 | -1.06 |
| PN_3_-G | 0.903 | -0.74 |

**Table 2** The O_2_ and CO adsorption energies (E_ads_, eV) on P-N_x_C_y_-G, their corresponding Mulliken charge (q, |e|) of O_2_/CO.

| **Surfaces** | **Adsorbate** | **E_ads_** | **q(P)** | **q(N)** | **q(C)** | **q(gas)** |
| --- | --- | --- | --- | --- | --- | --- |
| PC_3_-G | Bare | - | 0.638 | - | -0.334 | - |
|  | O_2_ (End-on) | -1.50 | 1.103 | - | -0.365 | -0.565 |
| PN_1_C_2_-G | Bare | - | 0.692 | -0.595 | -0.349 | - |
|  | O_2_ (End-on) | -0.54 | 1.382 | -0.642 | -0.406 | -0.614 |
|  | O_2_ (Side-on) | -1.76 | 1.401 | -0.652 | -0.388 | -0.685 |
|  | CO | 0.48 | 0.716 | -0.573 | -0.339 | 0.001 |
|  | O* | -4.83 | 1.196 | -0.722 | -0.362 | -0.541 |
| PN_2_C_1_-G | Bare | - | 0.810 | -0.601 | -0.371 | - |
|  | O_2_ (Side-on) | -2.83 | 1.532 | -0.653 | -0.403 | -0.674 |
|  | CO | -0.20 | 0.817 | -0.605 | -0.351 | 0.007 |
|  | O* | -4.53 | 1.332 | -0.737 | -0.376 | -0.522 |
| PN_3_-G | Bare | - | 0.903 | -0.612 | - | - |
|  | O_2_ (Side-on) | -4.58 | 1.348 | -0.645 | - | -0.592/-0.582 |
|  | CO | -0.23 | 0.909 | -0.617 | - | 0.008 |

**Table 3**. Activation energies of the rate-determining step for CO oxidation on some metal-free catalysts.

| **Catalyst** | **Rate determining step (eV)** |
| --- | --- |
| Si-C_3_N nanosheet [1] | 0.38 (LH) |
| B-N_3_ graphene [2] | 0.39 (ER) |
| N-doped / P-doped graphene [3] | 0.67 (LH)/ 0.54 (LH) |
| B-doped/B-N doped penta-graphene [4] | 0.34 (ER)/ 0.39 (ER) |
| P/FeP_4_/ CoP_4_/ NP_4_- doped graphene[5] | 1.19/0.87/0.81/0.79 (LH) |
| PN_1_C_2_-G /PN_2_C_1_-G | 0.60/0.26 (ER) (*the present study*) |
| PN_1_C_2_-G | 0.86 (LH) (*the present study*) |
| PN_1_C_2_-G /PN_2_C_1_-G | 1.46 /1.19 (NER)  (*the present study*) |

References

1 Esrafili, M. D. & Heydari, S. An effective approach for tuning catalytic activity of C3N nanosheets: Chemical-doping with the Si atom. *Journal of Molecular Graphics and Modelling* **92**, 320-328, doi:<https://doi.org/10.1016/j.jmgm.2019.08.011> (2019).

2 Esrafili, M. D. & Mousavian, P. Boosting graphene reactivity with co-doping of boron and nitrogen atoms: CO oxidation by O2 molecule. *Applied Surface Science* **455**, 808-814, doi:<https://doi.org/10.1016/j.apsusc.2018.06.053> (2018).

3 Esrafili, M. D., Mohammad-Valipour, R., Mousavi-Khoshdel, S. M. & Nematollahi, P. A Comparative Study of CO Oxidation on Nitrogen- and Phosphorus-Doped Graphene. **16**, 3719-3727, doi:<https://doi.org/10.1002/cphc.201500488> (2015).

4 Krishnan, R., Wu, S.-Y. & Chen, H.-T. Catalytic CO oxidation on B-doped and BN co-doped penta-graphene: a computational study. *Physical Chemistry Chemical Physics* **20**, 26414-26421, doi:10.1039/C8CP04745F (2018).

5 Xu, X.-Y., Lin, S., Xu, H., Guo, H. & Zhao, C. Mechanistic insights into the CO oxidation reaction catalyzed by P-coordinated metal-doped graphene: The roles of phosphorus and metal atom. *Applied Surface Science* **556**, 149776, doi:<https://doi.org/10.1016/j.apsusc.2021.149776> (2021).
